# LncRNA RP11-19E11 is an E2F1 target required for proliferation and survival of basal breast cancer

**DOI:** 10.1038/s41523-019-0144-4

**Published:** 2020-01-06

**Authors:** A. Giro-Perafita, L. Luo, A. Khodadadi-Jamayran, M. Thompson, B. Akgol Oksuz, A. Tsirigos, B. D. Dynlacht, I. Sánchez, F. J. Esteva

**Affiliations:** 1 Division of Hematology/Oncology, Perlmutter Cancer Center, NYU Langone Health, New York, NY USA; 2 Applied Bioinformatics Laboratories, NYU School of Medicine, New York, NY USA; 3 Department of Pathology, NYU School of Medicine, New York, NY USA; 4 Present Address: Program in Systems Biology, University of Massachusetts Medical School, Worcester, USA

**Keywords:** Breast cancer, Predictive markers, Non-coding RNAs, Tumour heterogeneity

## Abstract

Long non-coding RNAs (lncRNAs) play key roles in the regulation of breast cancer initiation and progression. LncRNAs are differentially expressed in breast cancer subtypes. Basal-like breast cancers are generally poorly differentiated tumors, are enriched in embryonic stem cell signatures, lack expression of estrogen receptor, progesterone receptor, and HER2 (triple-negative breast cancer), and show activation of proliferation-associated factors. We hypothesized that lncRNAs are key regulators of basal breast cancers. Using The Cancer Genome Atlas, we identified lncRNAs that are overexpressed in basal tumors compared to other breast cancer subtypes and expressed in at least 10% of patients. Remarkably, we identified lncRNAs whose expression correlated with patient prognosis. We then evaluated the function of a subset of lncRNA candidates in the oncogenic process in vitro. Here, we report the identification and characterization of the chromatin-associated lncRNA, RP11-19E11.1, which is upregulated in 40% of basal primary breast cancers. Gene set enrichment analysis in primary tumors and in cell lines uncovered a correlation between RP11-19E11.1 expression level and the E2F oncogenic pathway. We show that this lncRNA is chromatin-associated and an E2F1 target, and its expression is necessary for cancer cell proliferation and survival. Finally, we used lncRNA expression levels as a tool for drug discovery in vitro, identifying protein kinase C (PKC) as a potential therapeutic target for a subset of basal-like breast cancers. Our findings suggest that lncRNA overexpression is clinically relevant. Understanding deregulated lncRNA expression in basal-like breast cancer may lead to potential prognostic and therapeutic applications.

## Introduction

Breast cancer is the most frequently diagnosed malignancy in women worldwide and is the second leading cause of cancer-related death in the United States.^1^ Expression of the estrogen receptor (ER), progesterone receptor (PR), and amplification of the *HER2* gene define the main breast cancer subtypes in terms of prognostic and therapeutic intervention. Gene expression profiling based on complementary DNA (cDNA) microarrays led to the classification of breast cancer into distinct subtypes, with separate prognostic and treatment implications: luminal A (LA), luminal B (LB), basal-like (BL), and Her2-enriched (HER2).^2,3^ The PAM50 assay measures the messenger RNA (mRNA) expression levels of 50 genes that can classify breast cancers into the same subtypes. Triple-negative breast cancer (TNBC), defined as lacking expression of ER/PR/HER2 receptors, represents 15–20% of breast cancer, and it is associated with the highest probability of relapse among breast cancer subtypes despite local treatments and cytotoxic chemotherapy.^4^ The majority of TNBCs are classified as BL and vice versa, with an overlap between the two classifications of ~80%.^5^ The broad heterogeneity of TNBC, both inter- and intra-tumoral, has contributed to the difficulties in successfully treating it. Indeed, gene expression profiling performed in triple-negative breast cancers displayed six independent clusters with specific ontology, including two BL (BL1 and BL2), immunomodulatory (IM), mesenchymal, mesenchymal stem-like (MSL), and luminal androgen receptor (LAR)^6^ subtypes.

With the development and improvement of genomic sequencing with high-throughput technologies, we have learned that while most of the genome is transcribed (96–98%), ~2% of these transcripts encode for proteins.^7^ Although most of these non-coding transcripts have been considered junk DNA historically, in the past few decades, non-coding RNAs have been implicated in a variety of normal biological processes and disease states.^8,9^ Furthermore, the number of non-coding elements increases more rapidly than protein coding genes (PCG) with increasing organismal complexity.^10^ In addition, a high proportion of disease-related genetic variants identified with genome-wide association studies (GWAS) map to non-coding regions, suggesting a biological role for these transcripts in health and disease.^11^

Long non-coding RNAs (lncRNAs) are a large and diverse class of non-coding RNA transcripts with a length ≥200 nucleotides. LncRNA expression has been implicated in a variety of biological processes, ranging from development and cell cycle control to apoptosis and carcinogenesis.^8,9^ Emerging lncRNA functional mechanisms are diverse and versatile; lncRNAs may act as guides, decoys, or scaffolds for chromatin modeling complexes, regulate post-transcriptional mRNA decay, or act as sponges for miRNA and regulate mRNA splicing, among other functions.^12^ We and others have shown that the lncRNA landscape in breast cancer is subtype-specific. Using unsupervised clustering analysis, lncRNA expression can classify breast cancers similarly to PCG expression.^13,14^ Additionally, accumulating evidence indicates that several lncRNAs are involved specifically in breast carcinogenesis.^13,15^

In the present study, we sought to identify clinically relevant lncRNAs deregulated specifically in basal-like breast cancer patients and then functionally evaluated a subset of these candidates in the oncogenic process in vitro and assessed their value as prognostic markers. We identified and characterized the chromatin-associated lncRNA, RP11-19E11.1, which is upregulated in 40% of basal primary breast cancers. Gene set enrichment analysis (GSEA) in primary tumors and in cell lines uncovered a correlation between RP11-19E11.1 expression level and the E2F oncogenic pathway. We show that this lncRNA is chromatin-associated and an E2F1 target, and its expression is necessary for cancer cell proliferation. Finally, we used lncRNA expression levels as a tool for drug discovery in vitro and identified PKC as a potential therapeutic target for a subset of BL breast cancers.

## Results

### Identification of clinically relevant lncRNAs overexpressed specifically in BL breast cancer

**Fig. 1:**
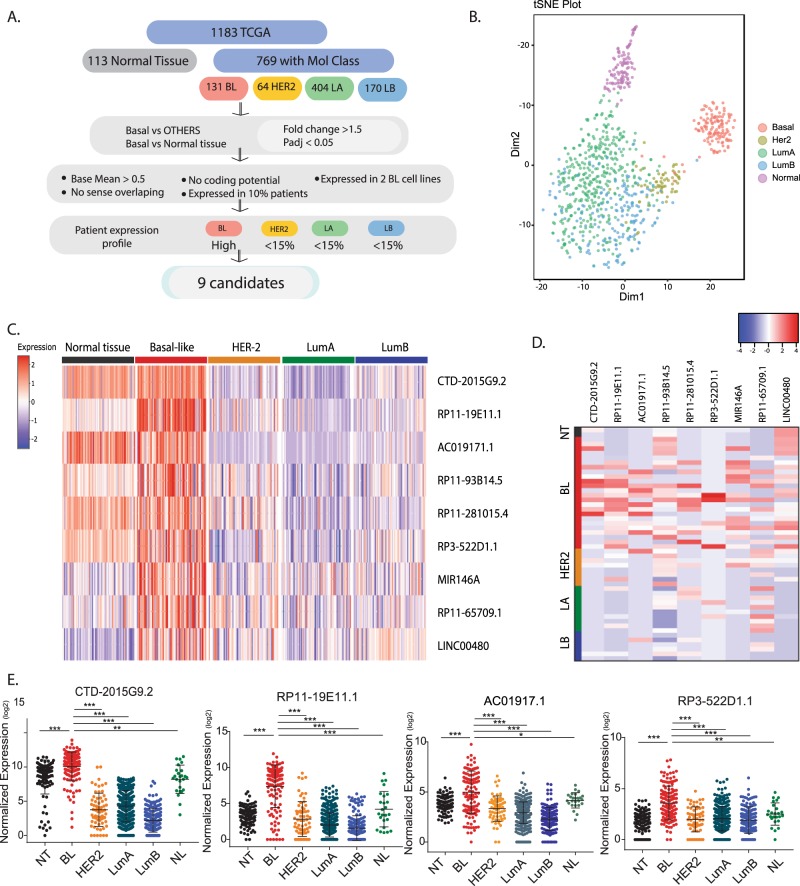
Identification of lncRNAs specific for the basal-like breast cancer subtype using patient data from the TCGA database. **a** Pipeline flow chart used to identify lncRNA candidates. **b** t-SNE plot originated from the expression of the 500 top-expressed lncRNA in patients. **c** Heatmap showing the expression of the nine lncRNA candidates in different breast cancer subtypes. **d** Heatmap showing the expression of the nine candidates in a panel of cell lines classified according to their molecular subtype. **e** Dot plot showing the levels of expression for each patient within the breast cancer subtypes for the lncRNA candidates selected. Mean ± SD are represented for each condition. *P < 0.05; **p < 0.01; ***p < 0.001 (ANOVA). NT normal tissue, BL basal-like, HER2 her2-enriched, LA luminal A, LB luminal B, NL normal-like.

In order to identify lncRNAs that play a role in BL breast cancer, we used RNA-sequencing (RNA-seq) data from 1183 patients available in the The Cancer Genome Atlas (TCGA) database. We classified the tumors with available PAM50 molecular subtype annotation,^16^ obtaining a final cohort of 769 patients represented by 131 BL, 64 HER2, 404 LA, and 170 LB subtypes (Fig. 1a). We excluded 25 tumors initially classified as normal-like (NL) subtype for further analysis. We and others have previously shown that lncRNA expression reflects subtype specificity in breast cancer.^13,14,17^ Accordingly, t-SNE (t-distributed stochastic neighbor embedding) analysis of the top 500 lncRNAs expressed in patients showed a molecular subtype-based clustering for this cohort of patients, similar to the one obtained using marker genes, a mix between coding and non-coding genes (Fig. 1b, Supplementary Fig. 1a). Using differentially expressed gene analysis (DEseq analysis), we identified a subset of lncRNAs overexpressed in the BL subtype (>1.5-fold change) compared to normal tissue and other subtypes. To enrich for clinically relevant lncRNAs, we filtered out those with low baseMean (<0.5) expressed in fewer than 10% of the patients. To study their function in vitro, we analyzed the RNA-seq data of a panel of cell lines and selected those lncRNAs that were expressed in at least two BL breast cancer cell lines. To further restrict the list of potential lncRNAs overexpressed in BL breast cancer, we selected the genes that were highly expressed in BL breast cancer and minimally expressed in the other subtypes (cut-off was <15% of patients with FPKM (fragments per kilobase of exon model per million reads mapped) >1) (Fig. 1c, d). Interestingly, this produced a list of nine candidates, expression of which was sufficient to cluster the BL subtype tumors by t-SNE analysis (Supplementary Fig. 1b).

### In vitro validation and functional screening of lncRNAs overexpressed in BL breast cancer

**Fig. 2:**
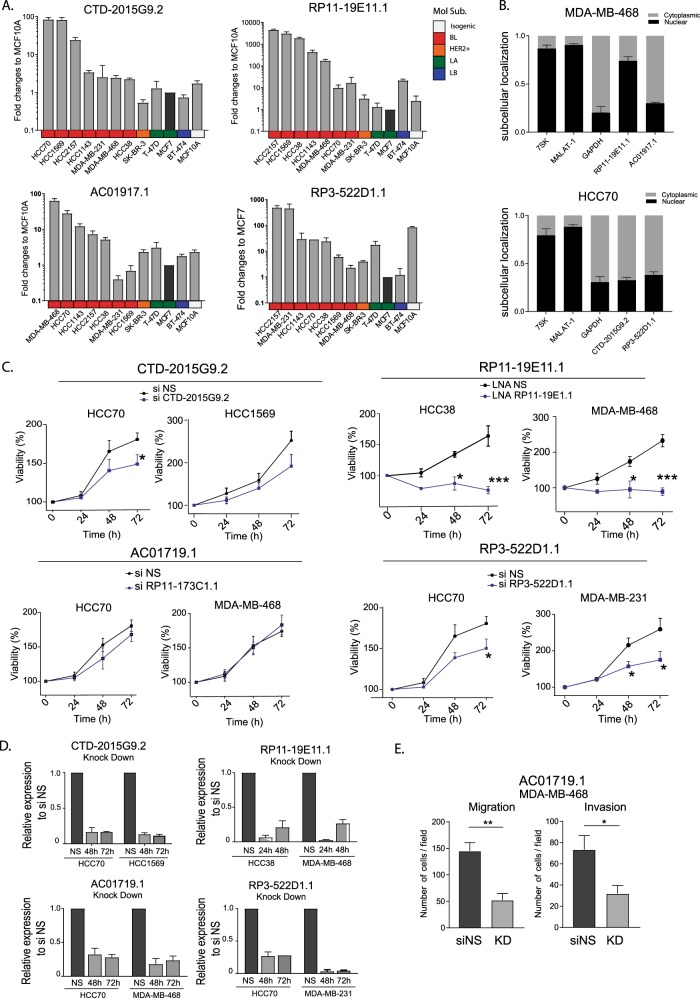
In vitro validation and functional screening of lncRNA candidates. **a** RNA expression obtained by qRT-PCR for four of the lncRNA candidates in a panel of cell lines. MCF7 cell line was used as reference. **b** Subcellular localization of the lncRNA candidates assessed by qRT-PCR after nuclear–cytoplasm fractionation. **c** Viability assays (MTT) after knock-down (siRNA 20 nM or LNA 50 nM) in two different cell lines for each of the lncRNA candidates at different time points. **d** RNA levels of the lncRNA candidates after knock-down in two cell lines at 48 and 72 h after transfection. **e** Migration and invasion in the MDA-MB-468 cell line after lncRNA AC01719.1 knock-down. Experiments were performed at least three times in triplicate. Data are presented as mean ± SEM. *P < 0.05; **p < 0.01; ***p < 0.001 (two-tailed unpaired *t* test).

To study potential roles of these lncRNAs in tumor growth or cancer progression using in vitro models, we analyzed their levels in cell lines representing different molecular subtypes of breast cancer with Quantitative reverse transcription PCR (qRT-PCR). We selected for further studies those lncRNAs showing detectable levels by qRT-PCR and overexpression (OE) of at least ten-fold in at least two BL cell lines compared to other subtypes of cancer and/or normal breast cell lines. LncRNAs CTD-2015G9.2, RP11-19E11.1, AC01917.1, and RP3-522D1.1 fulfilled these criteria, and since their expression patterns in cell lines mirrored those observed in patients, they were selected for further functional studies (Figs 1e and 2a). We next examined the localization of these lncRNAs as this could provide insight into their potential functions. Three of the four candidates showed mostly cytoplasmic localization (similar to glyceraldehyde 3-phosphate dehydrogenase (GAPDH)), while RP11-19E11.1 exhibited strong nuclear localization, similar to levels observed for nuclear non-coding RNA (ncRNA) controls, MALAT-1 and 7SK. (Fig. 2b). Using the patient survival data available in TGCA, we asked whether any of our candidates would behave as a prognostic marker. We divided the patients into high and low expression groups for each of the four candidates and performed log-rank test (Supplementary Fig. 2). Patients with higher levels of the lncRNA AC01917.1 had somewhat better survival profiles (*p* value 0.035), whereas patients with high levels of RP11-19E11.1 exhibited poor survival outcomes (*p* value 0.041). We then asked if any of these lncRNAs played a role in cancer cell growth and migration. To assess this, we designed small interfering RNAs (siRNAs) for the cytoplasmic lncRNAs and locked nucleic acid (LNA) GapmeRs (LNAs) to knock down the nuclear lncRNA. Initially, we measured cell viability after transcript knock-down using two different cell lines that overexpressed the lncRNA candidates (Fig. 2c, d). Of note, viability was reduced in all cell lines depleted of CTD-2015G9, RP11-19E11.1, or RP3-522D1.1, but there was no effect observed after knock-down of AC01917.1. We therefore tested whether this lncRNA might be important in cell migration and/or invasion (Fig. 2e) and found that knock-down of AC01917.2 in two different cell lines impaired migration and invasion in Boyden chamber migration assays.

Thus, our studies indicate that we have identified a small group of lncRNA candidates with BL subtype-specific expression in both patients and cell lines. Importantly, our ability to cluster BL tumors based on this profile suggests that these lncRNAs could have prognostic value and play a potential role in tumor development and carcinogenesis.

### Genetic characterization of the BL-specific and chromatin-associated lncRNA RP11-19E11.1

The nuclear lncRNA, RP11-19E11.1, is overexpressed in 40% of BL patients. We showed that cell viability was strongly impaired after knock-down and that patients with high levels of this lncRNA have a poor prognosis. Therefore, we sought to characterize its potential function in depth and focused on this lncRNA in the remainder of our study.

**Fig. 3:**
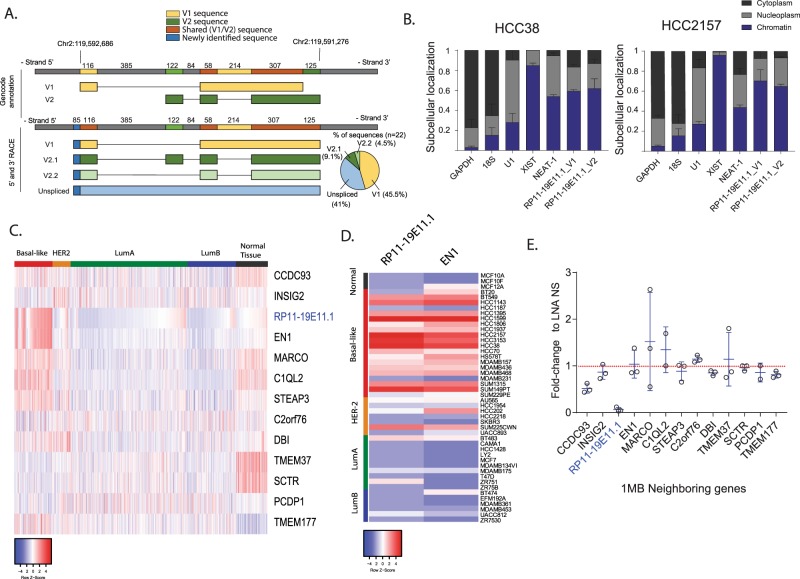
LncRNA RP11-19E11.1 transcript characterization. **a** Scheme representing 5′ and 3′ RACE results for the three main variants identified. **b** Subcellular localization of V1 and V2 assessed by qRT-PCR after nuclear–cytoplasm fractionation and chromatin precipitation. Data are presented as mean value ± SEM. **c** Heatmap of the expression of 1 Mb neighboring genes of lncRNA RP11-19E11.1 in TCGA patients. **d** Heatmap of the expression of RP11-19E11.1 and EN1 in a panel of cell lines. **e** Dot plot showing the expression levels of RP11-19E11.1 neighboring genes for each cell line. For each gene mean ± SD is represented. Value not shown for those genes in which expression was below detection level. Experiments were performed at least three times in triplicate.

RP11-19E11.1 is an intragenic lncRNA. The annotation for this lncRNA identified two variants of the transcript with different 5′ and 3′ ends (Fig. 3a). In order to verify the authentic 5′ and 3′ ends of this lncRNA, we performed 5′ and 3′ rapid amplification of cDNA ends (RACE) using the nuclear fraction of a cell line that highly expresses this lncRNA (HCC2157). Our results showed that in contrast with previous annotations, variants 1 and 2 (V1 and V2) share the same 3′ end (Fig. 3a). Further, we were not able to detect any transcripts with the 5′ end of annotated V2, while two different isoforms with the same introns as V2 shared the 5′ end of V1 (Fig. 3a). Sequencing results of the RACE clones showed that all the variants incorporated 85 additional nucleotides at the 5′ end of the annotated V1.

We next explored whether this lncRNA is associated with chromatin, as many nuclear lncRNAs have been reported to play a role in chromatin remodeling or transcriptional regulation, among other functions.^18,19^ Our results clearly indicated chromatin association, similar to Neat-1 or Xist, used as controls for both V1 and V2 (Fig. 3b). Nuclear lncRNAs can regulate expression of neighboring genes *in cis*. To investigate this hypothesis, we plotted a heatmap for the expression of all genes found in a 1 Mb window around the lncRNA (Fig. 3c). Strikingly, we observed a near-perfect correlation in expression between RP11-19E11.1 and EN1, a neural-specific transcription factor localized ~13 kb away that was implicated in basal-like breast cancer.^20^ This correlation was also observed in multiple cell lines (Fig. 3d). However, after knock-down of the lncRNA, we did not observe a significant change in the expression of neighboring genes, including EN1 (Fig. 3e). Moreover, knock-down of EN1 did not significantly change RP11-19E11.1 expression (Supplementary Fig. 3a). We also noted that OE of both transcripts was not due to an amplification of this region on the chromosome (cBioportal). Since both promoters of RP11-19E11.1 and EN1 are enriched for CpG islands, we speculated that the observed co-OE could be due to deregulation of DNA methylation in the promoter of these two transcripts. Using TCGA methylation data, we found that the percentage of methylated CpG islands in this region was significantly lower for triple-negative patients than the other subtypes of cancer or normal tissue (Supplementary Fig. 3b). DNA methylation is an essential layer of regulation in mammalian genomes, controlling several biological processes, including development. Patterns of DNA methylation are profoundly altered in cancer, both by suppressing the transcription of tumor suppressor genes by promoter hypermethylation and by activating gene transcription due to global hypomethylation.^21^ Our data suggest that expression of RP11-19E11.1 could be deregulated in a subset of basal tumors as a result of hypomethylation of a large chromosomal region that includes this lncRNA. Since all RP11-19E11.1 transcripts identified show nuclear localization and chromatin association, we propose that the function of this lncRNA is linked to regulation of chromatin-associated processes.

### Patients with high RP11-19E11.1 show specific oncogenic signature

**Fig. 4:**
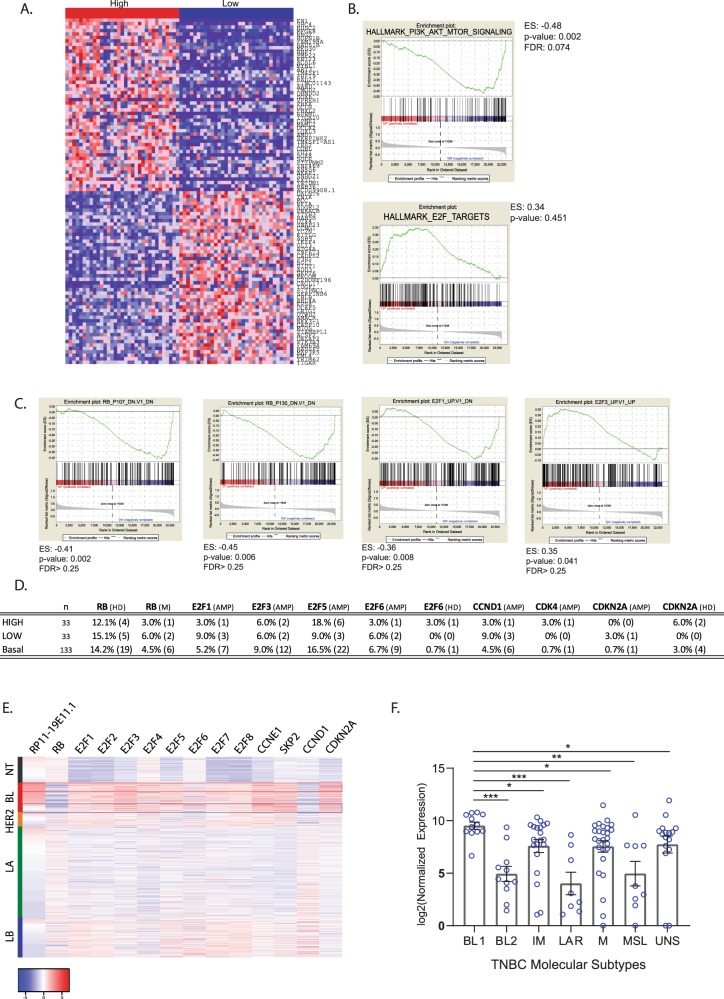
GSEA analysis of basal-like patients with high (*n* = 33) vs. low (*n* = 33) expression levels of RP11-19E11.1. **a** Heatmap of top and bottom ranked genes in patients with high and low RP11-19E11.1. **b** GSEA enrichment results for hallmark repository. **c** GSEA enrichment results for Oncogenic pathways. **d** Gene alteration in E2F axis in patients with high (*n* = 33) or low (*n* = 33) RP11-19E11.1 levels or within the basal-like subtype (labeled as basal). **e** Heatmap of E2F- and E2F-regulated genes in breast cancer subtypes. **f** Dot plot showing the expression of RP11-19E11.1 for each patient within the molecular subtypes identified by Vanderbilt classification in 102 TNBC patients from TCGA (Mann–Whitney test). Mean ± SEM is presented for each condition. *P < 0.05; **p < 0.01; ***p < 0.001. HD homodeletion, M mutation, AMP amplification.

Since patients with high levels of RP11-19E11.1 have a poorer prognosis than those exhibiting lower expression, we asked if patients that overexpress RP11-19E11.1 share a specific oncogenic signature. To that end, we compared BL patients with high and low RP11-19E11.1 expression (1Q vs. 4Q, *n* = 33 for each group) using GSEA (Fig. 4a). Intriguingly, patients with high levels of RP11-19E11.1 showed significant downregulation of the hallmark phosphatidylinositol-3-kinase/Akt/mammalian target of rapamycin signaling pathway and upregulation of the RB/E2F oncogenic pathway (Fig. 4b, c). We then evaluated if there was any alteration in some of the principal effectors of this axis commonly deregulated in cancer (RB, E2F, CDKN2A, CCDN1, CDK4) by amplification, deletion, or mutation that could be associated with the expression pattern of the lncRNA^22^ (Fig. 4d). Deregulation of this pathway is known to be prevalent in breast cancer and is connected to poor outcome.^23^ We did not find significant differences between patients with high and low RP11-19E11.1 in the prevalence of any gene alterations that can activate these pathways (Fig. 4d, e). As patients with high RP11-19E11.1 show a specific oncogenic signature, we analyzed whether the expression of this lncRNA was restricted to a unique TNBC molecular subtype, as defined by another well-established classification scheme.^6,24^ The stratification of 102 TNBC patients in these six different subtypes showed a significant enrichment of RP11-19E11.1 in the BL1 subtype (Fig. 4f). The BL1 is a subtype enriched in cell cycle and cell division components and pathways, showing increased proliferation and cell cycle check point loss. These results align well with our GSEA analysis. Furthermore, we also observed that this lncRNA was also highly expressed in the subtypes IM and mesenchymal, both subtypes having highly proliferative phenotypes compared to BL2, MSL, or LAR.

These results suggest that factors other than genetic background trigger the E2F oncogenic signature and that this signature correlates with poor prognosis in BL breast cancer.

### lncRNA RP11-19E11.1 knock-down induces cell cycle arrest and apoptosis

**Fig. 5:**
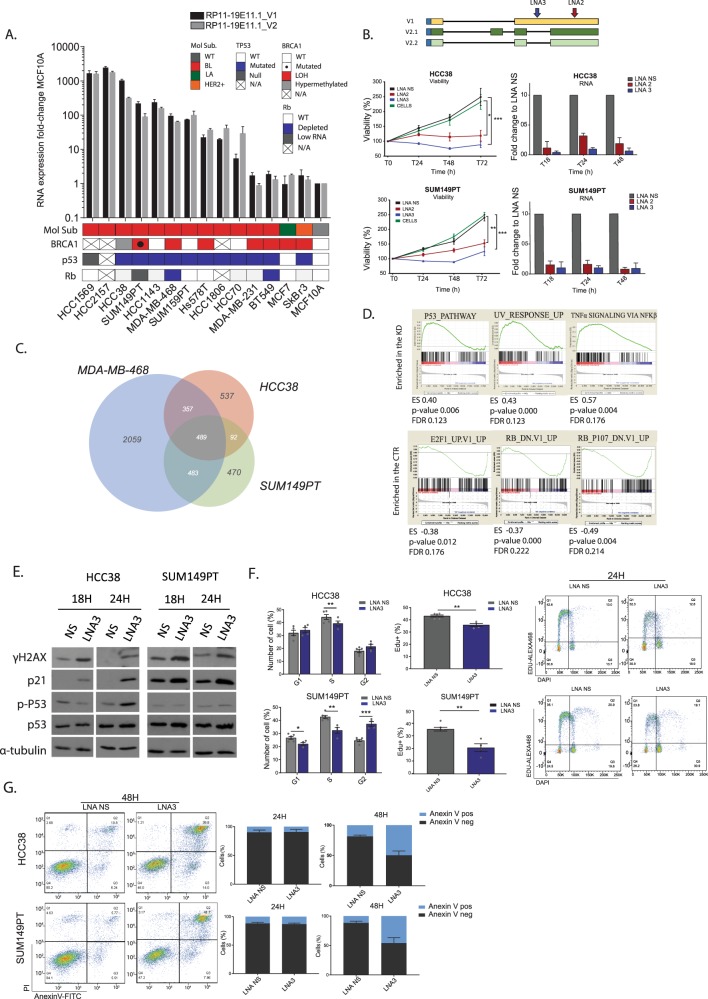
Functional characterization of lncRNA RP11-19E11.1. **a** RNA levels of both V1 and V2 assessed by qRT-PCR in a panel of basal-like cell lines. MCF10A was used as a reference. **b** Viability assay (MTT) in HCC38 and SUM149PT cell lines using two different LNAs with distinct target sites (LNA2/LNA3 25 nM). **c** Venn diagram of genes commonly deregulated (fold-change >2, false discovery rate (FDR) <0.1) in the three cell lines after 24 h of knock-down (LNA1 + LNA2 50 nM). **d** GSEA enrichment results for hallmark and oncogenic pathways after the knock-down of lncRNA RP11-19E11.1. **e** Protein analysis by WB of γH2AX, P53, p-P53, and p21 after 18 and 24 h after LNA3 transfection (25 nM). **f** Cell cycle analysis and EdU incorporation analysis (PI-EdU double staining) at 24 h after LNA3 transfection (25 nM) in HCC38 and SUM149PT cell lines. Each point represents a single measurement. **g** Annexin-V analysis after 24 and 48 h after LNA3 transfection (25 nM) in HCC38 and SUM149PT cell lines. Experiments were performed at least three times in triplicate. Data are presented as mean ± SEM. *P < 0.05; **p < 0.01; ***p < 0.001 (two-tailed unpaired *t* test).

We extended our analysis of a panel of BL cell lines to study the levels of both variants of the lncRNA RP11-19E11.1. We also performed viability assays (MTT, 3-(4-5-dimethylthiazol-2-yl)-2,5-diphenyl tetrazolium bromide) with two different LNAs separately, targeting different regions and variants of this lncRNA in two different cell lines, HCC38 (viability at 72 h for LNA2 vs. LNA NS *p* value 0.017, for LNA3 vs. LNA NS *p* value < 0.001) and SUM149PT (viability at 72 h for LNA2 vs. LNA NS *p* value 0.002, for LNA2 vs. LNA NS *p* value < 0.001) (Fig. 5a, b). We found that knock-down was most efficient 18 h after transfection (>90% for LNA3, ~80% for LNA2). In addition, we observed that both variants were down-regulated after knock-down, irrespective of which LNA was used, suggesting that they could target the unspliced transcript (Supplementary Fig. 4). We next performed RNA-seq after transfection of LNAs targeting RP11-19E11.1 in three cell lines. 489 genes were commonly deregulated in all three cell lines (fold-change >2 and false discovery rate <0.1, Fig. 5c). In order to identify the main pathways that may be linked to the lncRNA function, we performed GSEA analysis and evaluated enrichment of oncogenic pathways and transcription factor motifs in deregulated genes. Interestingly, our analysis showed that E2F/Rb oncogenic pathways and genes enriched for the E2F transcription factor motif were lost after RP11-19E11.1 knock-down (Fig. 5d). In contrast, gene sets that showed upregulation included the p53 pathway, ultraviolet (UV) response, and tumor necrosis factor-α signaling via nuclear factor-κB) (Fig. 5d).

In light of these transcriptomic changes, we analyzed DNA damage markers (γH2AX) and p53 pathway proteins by western blotting after RP11-19E11.1 knock-down. We observed substantial induction of γH2AX after 18 h. Also, p53 levels increased after the knock-down, in concert with its canonical target, p21/CDKN1A (Fig. 5e). Upregulation of other canonical p53 target genes, *GADD45* and *PMAIP1/Noxa*, was also observed after knock-down in both cell lines (Supplementary Fig. 5a). Accordingly, cell cycle analysis showed a concomitant decrease in the S-phase fraction in all cell lines (HCC38 *p* value 0.017, SUM149PT *p* value 0.002), and a G2/M phase block for SUM149PT cells (*p* value < 0.001), confirmed also by DNA synthesis inhibition measured by active 5-ethynyl-2′-deoxyuridine (EdU) incorporation (HCC38 *p* value 0.0011, SUM149PT *p* value 0.0014) (Fig. 5f). Given the apparent activation of p53 target genes and the DNA damage response, we asked whether apoptosis could be induced in cells depleted of this lncRNA. Indeed, apoptosis analysis using Annexin-V staining showed induction of cell death within 48 h of knock-down (Fig. 5g) in both cell lines.

These observations strongly connected our GSEA analysis with phenotypes described after RP11-1E11.1 knock-down, namely, E2F downregulation and cell cycle arrest, followed by activation of apoptotic pathways. We surmise that the resultant apoptosis could be due to replication stress or induction of the DNA damage response after RP11-19E11.1 knock-down.

### The induction of p53 downstream genes occurs in a partially p53-independent manner

p53 mutations have been reported in >80% of triple-negative breast cancers.^25^ Most of the mutations are localized to the DNA binding domain, perturbing the affinity of p53 for the promoter and altering the expression of transcriptional target genes.^26^ We noticed that HCC1569, a p53-null cell line, shows high levels of RP11-1911.1, and therefore we asked whether the knock-down of the lncRNA could induce the same response in p53 target genes. Unexpectedly, p21 levels were upregulated independently of p53 in this cell line (Supplementary Fig. 5b, c). Similar results were obtained with the p53-mutated SUM149PT cell line. The depletion of p53 in this cell line did not rescue cell viability after lncRNA knock-down, and levels of p21 increased both at the RNA and protein level independently of p53 protein levels (Supplementary Fig. 5b, d, e). Therefore, apoptosis induction can occur independently of p53 function, which is relevant for a subtype of breast cancer with a high frequency of p53 mutations.

### lncRNA RP11-19E11.1 is a E2F1 target gene

Patients with high levels of expression of RP11-1E11.1 have a specific gene signature related to activation of a subset of E2F target genes. Indeed, the loss of this lncRNA induces cell cycle arrest, thereby reducing proliferation and, subsequently, inducing apoptosis genes. Therefore, we examined three possible scenarios that which could link this lncRNA with the E2F gene signature. First, the lncRNA could control E2F1 transcription. Second, the lncRNA could be regulated by E2F transcription family members and therefore have an indispensable role in maintaining cell proliferation, or third, the lncRNA is involved in regulation of a specific subset of E2F downstream genes. The second and third possibilities are not mutually exclusive.

**Fig. 6:**
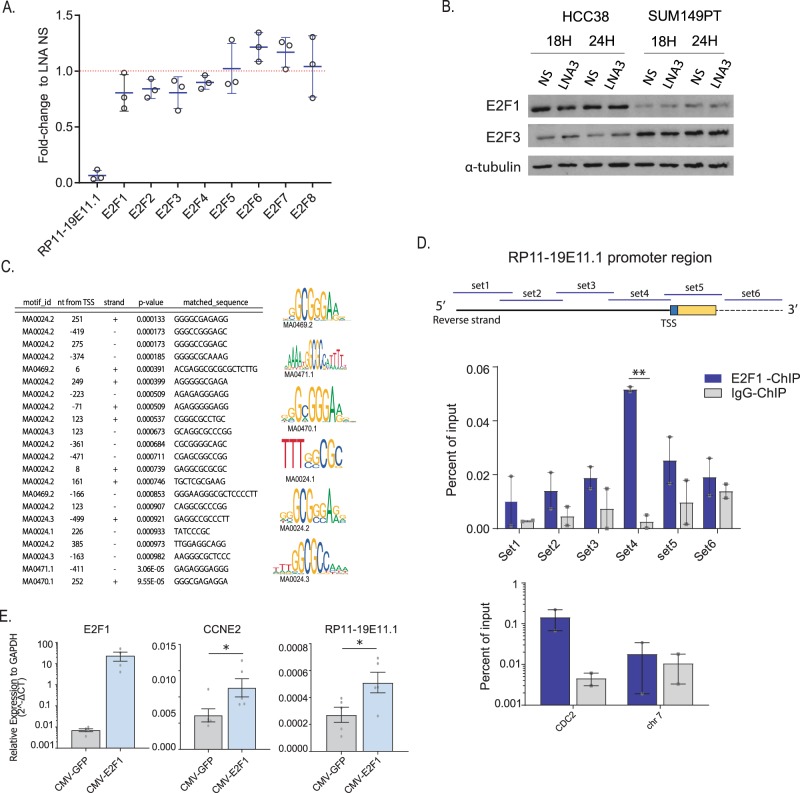
LncRNA RP11-19E11.1 is an E2F target gene. **a** RNA expression fold-change of E2F family transcription factors after RP11-19E11.1 knock-down from RNA-seq data. Each point represents the fold-change in each cell line, and for each gene mean value ± SD is represented. **b** Western blot for E2F1 and E2F3 after RP11-19E11.1 after 18 and 24 h using LNA3. **c** Motif enrichment analysis in the RP11-19E11.1 promoter for E2F transcription factor. **d** Chromatin immunoprecipitation (ChIP) using E2F1 antibody in HCC38. Two biological replicates are shown (two-tailed unpaired *t* test). **e** RNA levels of CCNE2 and RP11-19E11.1 in HCC38 and SUM149PT after exogenous overexpression of E2F1 (two-tailed paired *t* test). Each point represents a single measurement. Data are presented as mean ± SEM. *P < 0.05; **p < 0.01; ***p < 0.001.

To evaluate the first option, we checked the levels of the E2F family after RP11-1E11.1 knock-down using the RNA-seq data from three different cell lines. The results did not show significant changes in RNA in any of the E2F family members (Fig. 6a). Protein levels for E2F1 and E2F3 were also evaluated, and no significant changes were observed (Fig. 6b). Therefore, we next evaluated the possibility of the lncRNA being an E2F target gene. Promoter analysis identified several potential E2F motifs around the TSS (transcription starting site) of the lncRNA (*p* value < 0.001, Fig. 6c). To validate the hypothesis of a direct interaction of E2F1 with the RP11-19E11.1 promoter, we performed chromatin immunoprecipitation (ChIP) analysis. As several putative binding sites were identified, we designed six overlapping primers sets that spanned regions upstream and downstream of the TSS identified by 5′ RACE. Only primer set 4 (−141 + 13 around TSS) showed significant enrichment compared to the control (*p* value 0.003) (Fig. 6d). Concordantly, exogenous expression of E2F1 increased the expression of RP11-19E11.1 (*p* value 0.04) to an extent similar to another canonical E2F1 target gene, CCNE2 (*p* value 0.017) (Fig. 6e).

Finally, regarding the possibility of RP11-19E11.1 regulating E2F family target genes, we have shown in this study that RP11-19E11.1 depletion is followed by a reversal in the E2F gene signature in vitro, and a robust induction of DNA damage, block of DNA synthesis, and activation of apoptosis pathways. Therefore, we cannot exclude the contribution of an indirect downregulation of some E2F target genes due to a loss of cell fitness after RP11-19E11.1 depletion. Analyses beyond the scope of this report will help define the role of this lncRNA in the regulation of genes controlled directly or indirectly by the E2F transcription factor family.

E2F family members are well-known transcription factors that control several aspects of cell proliferation, from cell cycle progression to DNA damage checkpoints and repair. Several lncRNAs have been identified as E2F targets, including H19,^27^ ANRIL,^28^ and ERIC,^29^ and all of them have been shown to exhibit aberrant expression in tumor cells and to play important roles in cancer development. We propose that RP11-19E11.1 may be another member of this group of lncRNAs.

### LncRNA RP11-19E11.1 expression has predictive value for drug sensitivity in BL cell lines

**Fig. 7:**
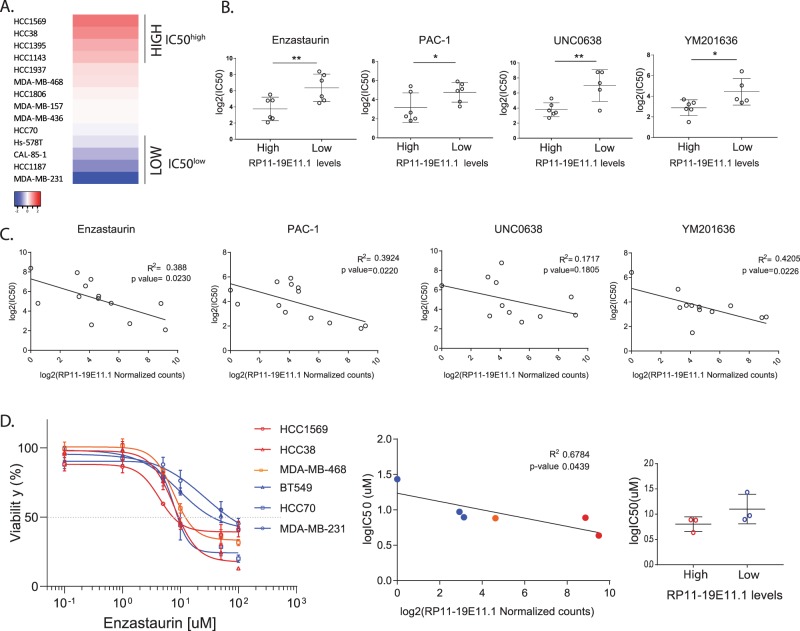
Drug screening using lncRNA RP11-19E11.1 levels as predictive value. **a** Basal-like cell lines with drug sensitivity data available ranked according to the expression of RP11-19E11.1 (obtained from CCLE.^31^) **b** Compounds identified with significantly different sensitivity (according to IC_50_ values) when comparing cell lines with low or high RP11-19E11.1 expressing levels. **c** Linear correlation between RNA expression and drug sensitivity (IC_50_) for the compounds identified in **b**. Pearson’s correlation test was used. CAL-85-1 cell line is not included (no expression data available). **d** Validation of the results for enzastaurin. Data are presented as mean ± SEM. *P < 0.05; **p < 0.01; ***p < 0.001 (unpaired *t* test unless specified).

Patients with high levels of the lncRNA show specific gene signatures within the BL subtype. We therefore asked whether drug sensitivity within the BL subtype could be predicted by the levels of expression of the lncRNA RP11-19E11.1. To that end, we explored the sensitivity of a selected panel of BL cell lines with different levels of expression of the lncRNA to more than 267 compounds available from The Genomics of Drug Sensitivity in Cancer.^30^ We found drug sensitivity screening data for 14 BL cell lines and overlapped these data with the respective expression levels of the lncRNA obtained from the RNA-seq data available in the CCLE.^31^ We ranked the four top and four bottom cell lines according to the expression of RP11-19E11.1 and compared the average half-maximal inhibitory concentration (IC_50_) of the high expressing cell lines to the average IC_50_ of the lowest ones (Fig. 7a). We selected for further analysis those drugs that showed an IC_50_^high^/IC_50_^low^ ratio <0.25, to ensure biological significance in drug sensitivity between groups. For those compounds that showed an IC_50_ significantly different when comparing the six top/bottom cell lines according to the lncRNA expression, we analyzed if there was a correlation between drug sensitivity and levels of expression of the lncRNA (Fig. 7b, c). Interestingly, three of the compounds showed a significant negative correlation between expression levels and sensitivity: PAC-1 (*p* value 0.022), enzastaurin (*p* value 0.023), and YM201636 (*p* value 0.023) (Fig. 7c). PAC-1 is the first pro-caspase activator compound developed and was designated as an orphan drug by the Food Drug Administration in 2016.^32^ Enzastaurin is an inhibitor of the protein kinase C (PKC), which failed the phase III treatment for the treatment of lymphoma.^33^ YM201636, is a PIKfyve (phosphatidylinositol phosphate kinase PIP5KIII) inhibitor, a kinase implicated in PtdIns(3,5)P2 biosynthesis that regulates a number of intracellular membrane trafficking pathways. Its inhibition disrupts endomembrane transport and retroviral release from infected cells.^34^

To validate the compounds identified in this screening, we selected three top/bottom cell lines after ranking our qRT-PCR results for RP11-19E11.1 levels. Time of treatment and viability assessment by metabolic assays were performed using methods similar to those employed in the screening. We found that neither PAC-1 nor YM201636 showed the tendency observed in the screening for the cell lines tested (Supplementary Fig. 6a, b). Enzastaurin, on the other hand, showed the expected results in all cell lines tested, except for SUM149PT (Supplementary Fig. 6c). Therefore, we decided to include another cell line with moderate levels of RP11-19E11.1, MDA-MB-468. Here, we reproduced the results observed in the screening, as a significant negative correlation (*p* value 0.044) was observed between RP11-19E11.1 levels and sensitivity to enzastaurin (Fig. 7d). IC_50_ values showed the same tendency observed in the screening but was not significant.

These results are quite provocative, as PKC activity leads to the activation of several pathways that can modulate a number of cellular processes, including proliferation and anti-apoptotic signaling.^35^ Among others, PKC can activate Raf1 by direct phosphorylation that subsequently can lead to phosphorylation of Rb protein, releasing E2F transcription factors.^36^ In addition, the disruption of Raf1-Rb protein interaction leads to tumor growth and angiogenesis inhibition.^37^ Furthermore, PKC can also activate Ras, which can induce E2F1 expression.^38^ Therefore, PKC inhibition could be an attractive target for further exploration in the treatment of basal breast cancer patients with an E2F activation signature.

## Discussion

Several studies have shown that lncRNA expression can be used to classify breast cancer into subtypes as effectively as PCGs,^13,14,39,40^ providing additional prognostic and predictive value.^41^ We identified a cohort of clinically relevant lncRNAs that are overexpressed in basal-like breast cancer as compared to normal tissue and other breast cancer subtypes, and we suggest that this signature may be useful in future studies aimed at understanding the etiology and treatment of this aggressive tumor.

The consequences and mechanisms of lncRNA dysregulation in basal-like/TNBC subtypes are not well defined. Two studies compared expression levels between TNBC vs. normal tissue using lncRNA microarrays in a small cohort of patients,^42,43^ while another study focused on breast cancer subtypes but did not take into account lncRNA expression in normal tissue,^40^ as we have done in our study. Furthermore, our studies are novel because we intersected patient data with cell line RNA-seq data in an effort to use clinical findings in in vitro studies for further functional and mechanistic studies. Interestingly, two of our candidates have been identified as basal-specific lncRNAs in previous studies, the CTD-2015G9.2 and RP11-19E11.1^40,44^ Another candidate that we identified is MIR146, the lncRNA host gene for mir-146a, a microRNA extensively studied and shown to play a role in TNBC/basal-like breast cancer.^45^ We validated lncRNA expression using qRT-PCR to confirm the expression pattern reported by RNA-seq in different cell line subtypes. Furthermore, we demonstrated that lncRNA ablation exerted a major impact on cell proliferation and migration/invasion, principal characteristics of the oncogenic signature.

We selected the lncRNA RP11-19E11.1 for further study in detail because its OE correlates with poor prognosis in patients with basal-like breast cancer. Importantly, its depletion impaired cell proliferation and strongly induced apoptosis. While our manuscript was in preparation, another group identified RP11-19E11.1 as a specific basal-like transcript in an independent cohort of tumor samples.^44^ In that study, the authors showed that this lncRNA was regulated through epigenetic modifications, in agreement with our observations, but no detailed description of the transcript, compartment localization, or putative function was provided. Here, we identified and characterized at least three RP11-19E11.1 transcript variants, correcting the actual Gencode annotation. Importantly, we also revealed that the two main lncRNA variants were chromatin-associated. Furthermore, by using GSEA analysis in patients and in cell lines, we linked the lncRNA expression to an E2F signature, demonstrating its connection to E2F experimentally by ChIP. We also provided transcriptome-wide analysis for three different cell lines after RP11-19E11.1 knock-down, further strengthening the connections between ablation of this lncRNA, activation of the DNA damage response, and induction of apoptosis. Therefore, our work has substantially expanded upon previous results, providing a better understanding of the role of RP11-19E11.1 in basal-like breast cancer.

Basal-like breast cancers are generally poorly differentiated tumors, are enriched in embryonic stem cell signatures, and show activation of proliferation-associated factors.^46^ The E2F transcription factors are well-known downstream effectors of the RB tumor suppressor and have a pivotal role in regulating cell cycle progression.^47,48^ They not only transcribe the subset of genes necessary for the transition from G1 to S phase, but they also have a role in controlling mitosis, DNA damage checkpoints and repair, and apoptosis.^47,48^ E2Fs are known to affect cancer development,^22^ and Rb dysfunction in TNBC is estimated to occur in ~30% of the cases.^49^ The influence of the E2F transcription factors in breast cancer development is undisputed.^23^ In this study, we identified an E2F oncogenic signature in patients with upregulated RP11-19E11.1 levels. Furthermore, our results showed RP11-19E11.1 to be an E2F1 target and a chromatin-associated lncRNA. Indeed, knock-down of RP11-19E11.1 induced robust inhibition of DNA synthesis and reduced expression of E2F target genes, which was followed by a strong activation of DNA damage response and apoptotic pathways. We showed that these effects could occur independently of p53. Altogether, these results suggest that RP11-19E11.1 function could be linked to that of E2F. Several examples of lncRNAs controlling cell cycle can be found in the literature.^50^ For instance, *ANRIL*, an E2F1 target gene, promotes gene silencing by recruiting repressors to the *INK4* promoter. Another E2F1 target gene, H19/miR-67, seems to be involved in pRB pathway inactivation. Therefore, in order to elucidate the possible scenarios in which RP11-19E11.1 could be involved in regulating cell cycle, future studies should investigate RNA–DNA and RNA–protein interactions.

Patients with elevated RP11-19E11.1 expression shared a specific oncogenic signature linked to poor prognosis. We speculated that these patients would also show different sensitivities to drug treatment. Taking advantage of drug screening published data in cell lines, we identified a PKC inhibitor as a potential compound for further study. Notably, PKC not only shares a connection to E2F activation but is also involved in key steps of viral replication.^51^ The idea of virus-initiated breast cancer has been proposed but is controversial. More compellingly, a recently published study found that the expression of human endogenous retrovirus-K is strongly associated with the basal-like breast cancer, and a strong association with RB phosphorylation and cell cycle activation was observed in these patients.^52^

In conclusion, this study identifies clinically relevant basal-like lncRNAs using a large cohort of samples from TCGA. Some of the candidates identified may have prognostic value and may be directly implicated in the oncogenic phenotype. Finally, we characterized the lncRNA, RP11-19E11.1, identifying a novel chromatin-associated and E2F target lncRNA. Our results show that this lncRNA is necessary for cell cycle progression, and its ablation impairs cell viability by inducing apoptosis. We also identified an E2F-specific signature linked to the expression of RP11-19E11.1, and have used cell lines expressing this lncRNA to identify possible drug therapies, which could be tested in TNBC patients.

## Methods

### TCGA data

The read count tables for all the sequencing samples (*n* = 1162) in TCGA were downloaded from The National Cancer Institute’s Genomic Data Commons (GDC). The count tables were normalized based on their library size factors using DEseq2, and differential expression analysis was performed. Clustering was performed using iCellR (https://github.com/rezakj/iCellR) by selecting the dispersed genes running principal component analysis and t-SNE. All the methylation *β* values for the samples with Illumina 450 methylation array data (*n* = 892) were downloaded from GDC.

### RNA-seq data processing

All the raw sequencing reads from our samples (Gene Expression Omnibus (GEO) https://identifiers.org/geo:GSE138606 (2019)) and the cell lines downloaded from the GEO repository, https://identifiers.org/geo:GSE73526 (2016) and https://identifiers.org/geo:GSE48213 (2013), were mapped to the human reference genome (GRCh37/hg19) using the STAR aligner (v2.5.0c)^54^. Alignments were guided by a Gene Transfer Format file. The mean read insert sizes and their standard deviations were calculated using Picard tools (v.1.126) (http://broadinstitute.github.io/picard). The read count tables were generated using HTSeq (v.0.6.0)^55^ normalized based on their library size factors using DEseq2^56^ and differential expression analysis was performed. The read per million normalized BigWig files were generated using BEDTools (v.2.17.0)^57^ and bedGraphToBigWig tool (v.4). All downstream statistical analyses and generating plots were performed in R environment (v.3.1.1) (http://www.r-project.org/).

### Cell culture

Breast cancer cells MDA-MB-231, Hs578T, MDA-MB-157, MDA-MB-468, MCF7, SkBr3, and BT474 were cultured in Dulbecco’s modified Eagle’s mMedium: Nutrient Mixture F12 (DMEM/F12) (Corning) supplemented with 10% fetal bovine serum (FBS), P/S (50 U/mL), and l-Glut (1%). SUM149PT and SUM159PT were cultured with DMEM/F12 supplemented with 5% FBS, P/S (50 U/mL), and l-Glut (1%), insulin (5 µg/mL), and hydrocortisone (1 µg/mL). Breast cancer cells HCC2157, HCC38, HCC2157, BT549, HCC70, HCC1143, and T-47D were cultured in RPMI medium supplemented with 10% FBS, P/S (50 U/mL), and l-Glut (1%). Normal breast MCF10A cells were cultured in DMEM/F12 (Corning) medium supplemented with 5% horse serum, epidermal growth factor (20 ng/mL), cholera toxin (100 ng/mL), insulin (0.01 mg/mL), and hydrocortisone (500 ng/mL). All cells were cultured at 37 °C with 5% CO_2_. All cell lines were obtained from ATCC, except from SUM149PT and SUM159PT (gifted from Neel BG laboratory).

To ensure mycoplasma-free culture, cells were tested periodically using the Universal Mycoplasma Detection Kit (ATCC, 30-1012K).

### RNA interference (siRNA and LNA) Protocol

siRNA at 20 nM (Dharmacon) or Antisense LNA GapmeRs at 25/50 nM (Qiagen) transfection mix was prepared in OptiMEM (Gibco) and RNAiMAX at 2.5 µL/mL (Invitrogen) for 10 min at room temperature. Transfection mix was then added to the cells at the time of seeding. After 6 h, the media were replaced for fresh new media. siRNA and LNAs sequences are in Supplementary Methods.

### Cell transfection protocol

Cells were seeded in 6-well plates at a confluence of 20–30%. Next day, transfection mix was prepared using FuGene HD reagent (Promega) at 3:1 ratio with 1 µg of pCMV-E2F1 or pCDH-EGFP in OptiMEM. After 10 min, the mixture was added to the cells with a final volume of 1 mL in 0.5% DMEM/F12 media. After 6 h, one volume of 10% FBS media was added. Next day, transfection media was replaced for fresh media and cells were collected for analysis after 48 or 72 h.

### Cell fractionation

Nuclear/cytoplasmic fractionation was performed as described.^53^ Briefly, cells were rinsed three times with cold phosphate-buffered saline (PBS) and scraped carefully. After spinning, disruption buffer (KCl 10 mM, MgCl_2_ 1.5 mM, Tris-Cl, pH 7.5, 20 mM, and dithiothreitol (DTT) 1 mM) was added and let to stand for 10 min. A type B Dounce was used to disrupt the cytoplasm using 10–15 strokes. When ~90% of the cytoplasm was broken, 0.1% of Triton X was added, mixed by inversion five times, and centrifuged for 5 min at 1500 r.p.m. The supernatant containing the cytoplasmic fraction was recovered by this method. Both supernatant and nuclei (pellet) were processed for RNA extraction using the SurePrep Nuclear or Cytoplasmic RNA Purification Kit (Fisher, BP2805-50) following user manual instructions. DNAaseI treatment was performed for both fractions (Norgen Biotek cat. num. 25710).

### Chromatin precipitation

Cell fractionation was performed as described above. The pellet containing the nuclear fraction was then resuspended in 125 μL of cold NUN1 buffer (20 mM Tris-HCl, 75 mM NaCl, 0.5 mM EDTA, 50% glycerol) and transferred to a new Eppendorf. Then, 1.2 mL of cold NUN2 buffer (20 mM HEPES-KOH, pH 7.6, 300 mM NaCl, 0.2 mM EDTA, 7.5 mM MgCl_2_, 1% NP40, 1 M urea) was added and vortexed. Samples were kept on ice for 15 min, vortexing every 3–4 min. Chromatin was recovered by centrifuging at 16,000 × *g* for 10 min at 4 °C. RNA was extracted from the supernatant fraction using TRIzol LS (Invitrogen) and for the chromatin fraction with TRIzol (Invitrogen).

### Reverse transcription and quantitative real-time PCR

Total RNA was isolated using the TRIzol reagent (Fisher). RNA was quantified with NanoDrop (Thermo Scientific) and RNA was reverse transcribed using Verso cDNA Synthesis Kit (Thermo Scientific). Quantitative reverse transcription PCR was performed using CFX96 Touch Real-Time PCR Detection System (Bio-Rad). Relative quantity of expression was calculated with the ΔΔCt method using GAPDH as an internal control. Primer sequences are in Supplementary Methods.

### Protein extraction and Western blot

Cells were trypsinized and washed with PBS. The pellet was then resuspended in fresh ELB buffer (50 mM HEPES, pH 7, 150 mM NaCl, 5 mM EDTA, 0.1% NP40, 10% glycerol) supplemented with a protease and phosphatase inhibitor cocktail (1 mM DTT, 0.5 mM AEBSF (4-(2-aminoethyl)benzenesulfonyl fluoride hydrochloride), 2 μg/mL leupeptin, 2 μg/mL aprotinin, 10 mM NaF, 50 mM β-glycerophosphate). Twenty micrograms of protein was loaded in a 10–15% acrylamide gels. Primary antibodies were diluted in 3% bovine serum albumin TBS-T (Tris-buffered saline with Tween-20) and incubated O/N at 4 °C. The primary antibody used and dilutions are α-tubulin (Sigma T5168, 1:5000), E2F1 (CST #3742, 1:1000), E2F3 (GeneTex GTX11843, 1:2000), p21 (CST #2947, 1:1000), P53 (Santa Cruz FL-393, 1:1000), p-P53 (CST #9286, 1:1000), γH2AX (CST #9718, 1:1000). All blots were derived from the same experiment and were processed in parallel. Un-cropped blots can be found in the Supplementary information file.

### FACS analysis

For EdU and cell cycle analysis, Click-iT EdU Alexa Fluor 488 Kit was used (Invitrogen C10337) following the manufacturer’s instructions.

Annexin-V staining was performed using Annexin-V Kit (Mileny Biotec, 130-092-052) following the manufacturer’s instructions. All analyses were performed with LSRII UV cell analyzer (BD Bioscience) and FlowJo software.

### Viability assays and compounds

For drug sensitivity evaluation, cells were seeded in a 96-well plate at 4000 cells/well. The next day, cells were incubated at 37 °C with different concentrations of the test compounds. Control cultures were incubated with dimethyl sulfoxide (DMSO). After 48 or 72 h, media were replaced with fresh media supplemented with MTT (Sigma) at a final concentration of 0.5 mg/mL. After 1.5 h, media were removed and formazan crystals were dissolved with 100 μl of DMSO. Absorbance at 570 nM was read in the spectrophotometer (TECAN Infinite M200). The drugs used in this assay were PAC-1 (S2738, Selleckchem), YM201636 (ref. 13576, Cayman Chemical), and enzastaurin (ref. HY-10342, MedChemExpress).

For viability assays after RNA interference, cells were seeded at 4000 cells/well and transfected at the same time as explained in the previous section. After 6 h, media were replaced with fresh new media. MTT assay was performed as explained above each day and viability was compared to T0. All experiments were repeated at least three times with different cell passage number, with three replicates per experiment.

### Rapid amplification of cDNA ends

Nuclear RNA was isolated from HCC2157 cells lines following the protocol described above. RACE was performed using the SMARTer RACE 5′/3′ Kit (Clontech) as per the manufacturer’s instructions. Primers were designed at known regions of the transcripts in order to achieve accurate 5′ or 3′ ends (Supplementary Methods).

### Chromatin immunoprecipitation

ChIP was performed following the previous described method with some modifications^58^. Nuclei were obtained from 1 × 10^7^ HCC38 cells and cross-linked with 1% formaldehyde. Sonication was performed with Branson Sonifier 450 on ice to obtain an average DNA length of 400–600 bp. The equivalent of 25 µg DNA was used per ChIP reaction. Chromatin was pre-cleared with protein A and then incubated with 2 μg of each antibody, E2F1 and IgG (Millipore #17-10061 Crosslinks) O/N. Chromatin was resuspended in 200 μL of 10 mM Tris, pH 8.0 buffer, and enrichment was assayed by quantitative PCR. Primers used are described in Supplementary Methods.

### Reporting summary

Further information on research design is available in the Nature Research Reporting Summary linked to this article.

## Supplementary information

Supplementary Information

Reporting Summary Checklist

## Data Availability

The raw RNA-sequencing data (supporting Fig. 1) used during the study are publicly available in the NCBI Gene Expression Omnibus (GEO) repository: https://identifiers.org/geo:GSE73526 and https://identifiers.org/geo:GSE48213. The read count tables (Supporting Figs 1–4) for all the sequencing samples (*n* = 1162) in TCGA are publicly available at The National Cancer Institute’s (NCI) Genomic Data Commons (GDC) (https://portal.gdc.cancer.gov/). RNA-sequencing datasets (Supporting Figs 3–6) generated during the current study are publicly available in the GEO repository: https://identifiers.org/geo:GSE138606. The Drug sensitivity in Breast Cancer Cell lines data (Supporting Fig. 7) are publicly available at https://www.cancerrxgene.org/downloads/drug_data?tissue=BRCA. Additional datasets used and generated during the study will be made available on request from the corresponding author, Prof. Francisco J. Esteva, as described in the following metadata record: 10.6084/m9.figshare.10266527^59^.

## Code availability

The code used in the present study is available at https://github.com/rezakj/iCellR.
