# Supplementary material for: LncRNA RP11-19E11 is an E2F1 target required for proliferation and survival of basal breast cancer
Source: NPJ Breast Cancer. 2020 Jan 6;6:1. doi: 10.1038/s41523-019-0144-4 (PMC6944689; doi:10.1038/s41523-019-0144-4)

Supplementary Figure 1

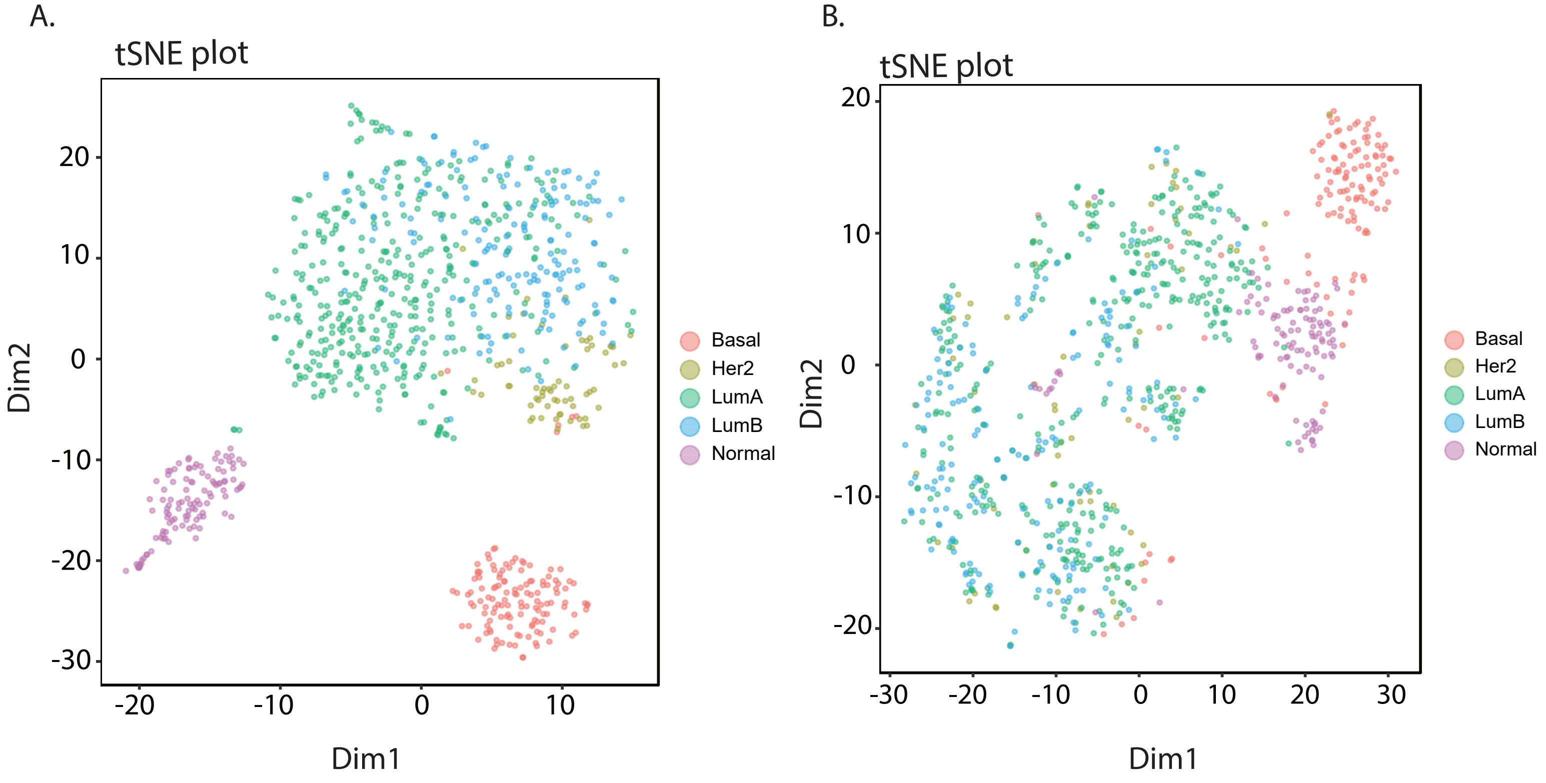

Supplementary Figure 1. T-SNE plots of TCGA patients using **A)** marker genes **B)** 9 lncRNA candidates.

Supplementary Figure 2

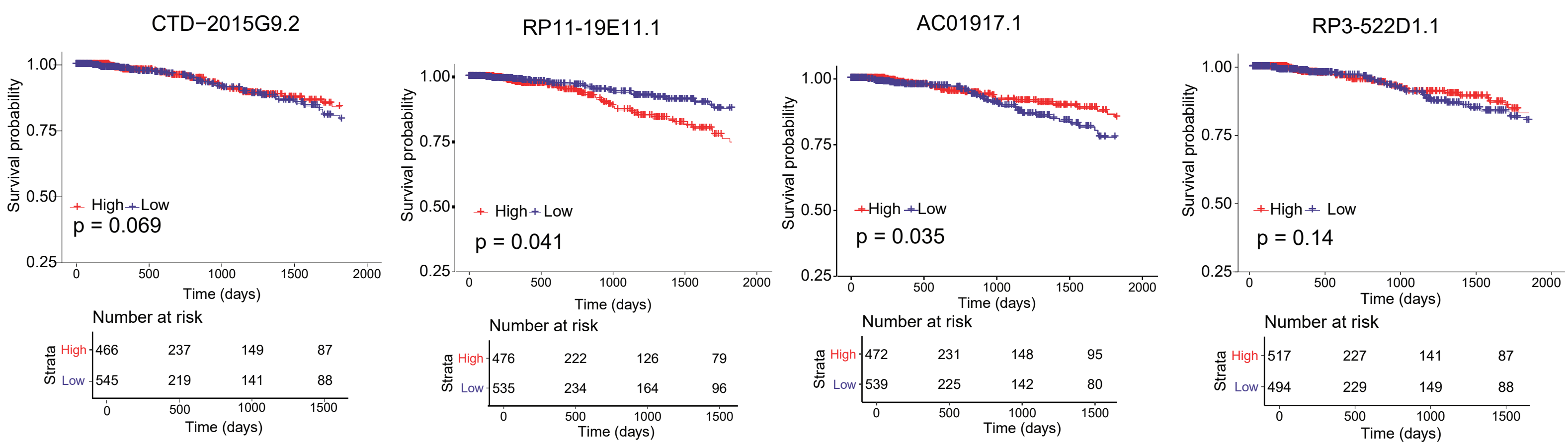

Supplementary Figure 2. Survival curves and log-Rank test for the lncRNA candidates selected for further functional studies. Median expression of the lncRNA was used for the stratification between low and high expression in patients.

Supplementary Figure 3

A.

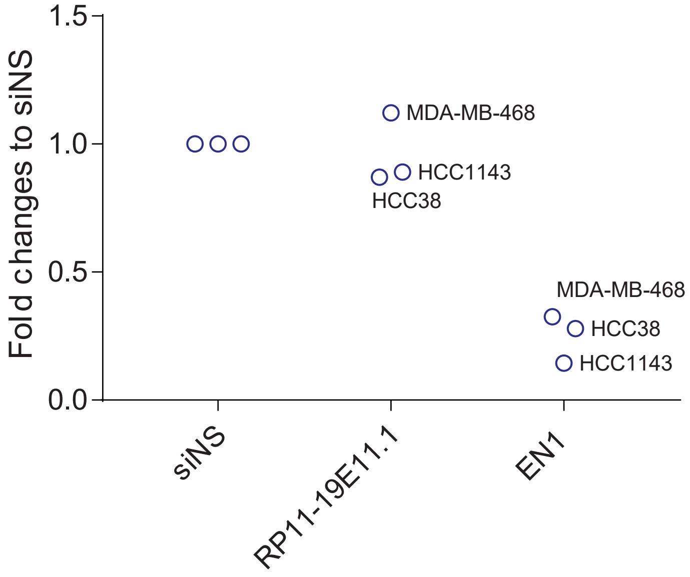

B.

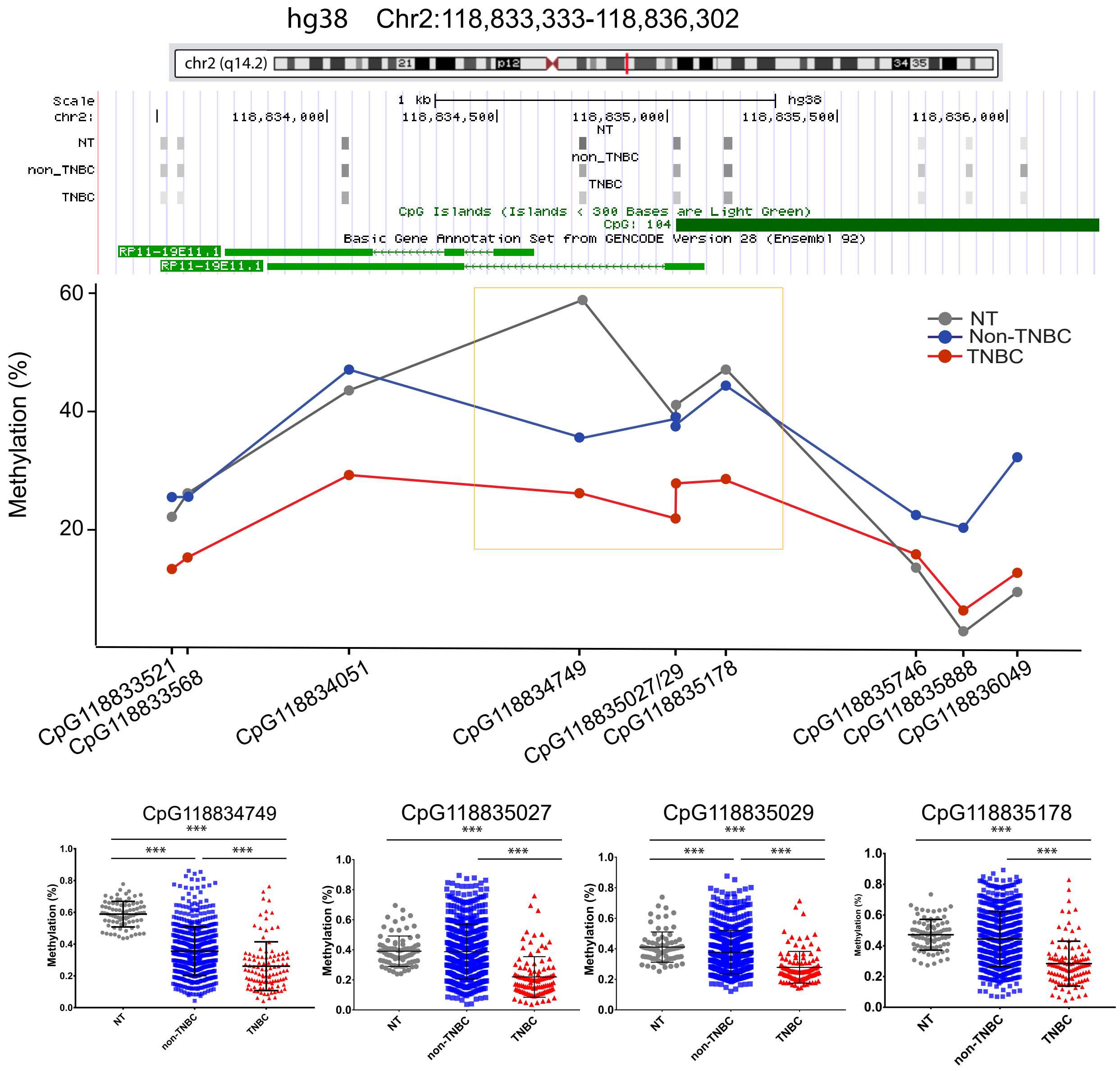

Supplementary Figure 3. RP11-19E11.1 characterization. **A)** RP11-19E11.1 expression levels after EN1 Knock-down in three different cell lines. **B)** DNA Methylation profile of RP11-19E11.1 promoter in Normal tissue, TNBC and non-TNBC.

Supplementary Figure 4

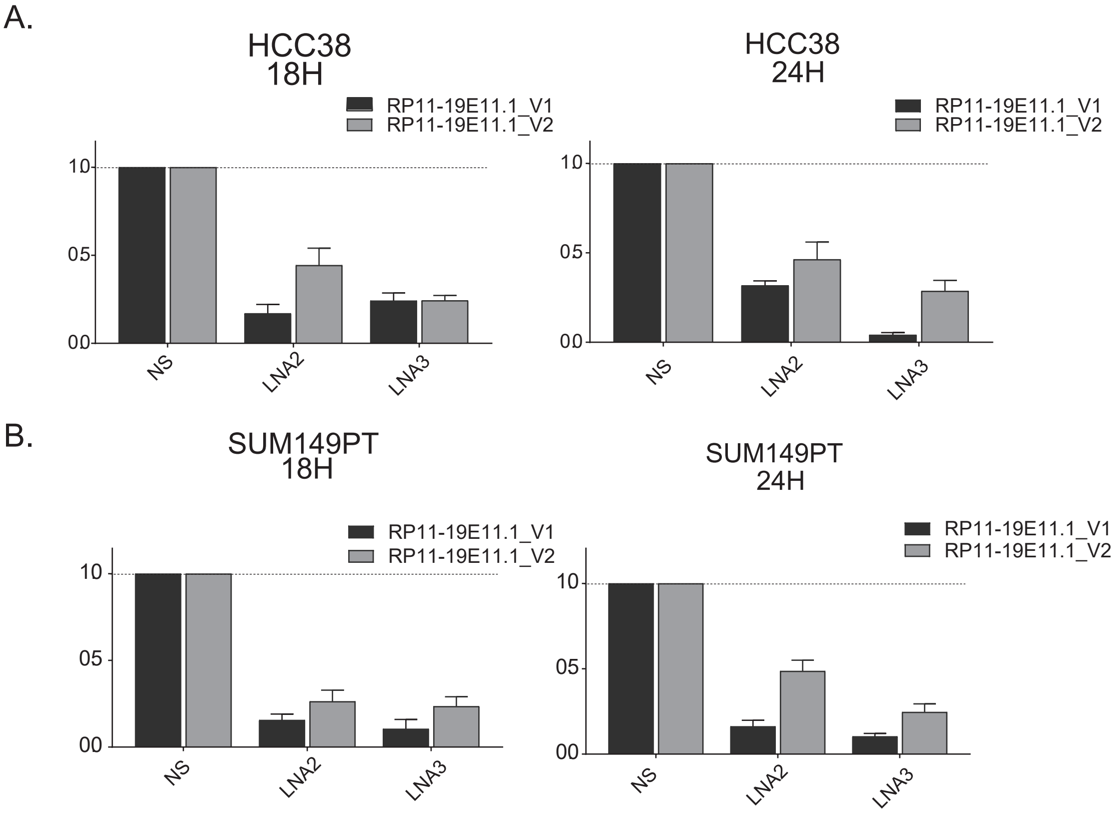

Supplementary Figure 4. RNA levels of V1 and V2 using two different LNA sequences for **A)** HCC38 and **B)** SUM149PT.

Supplementary Figure 5

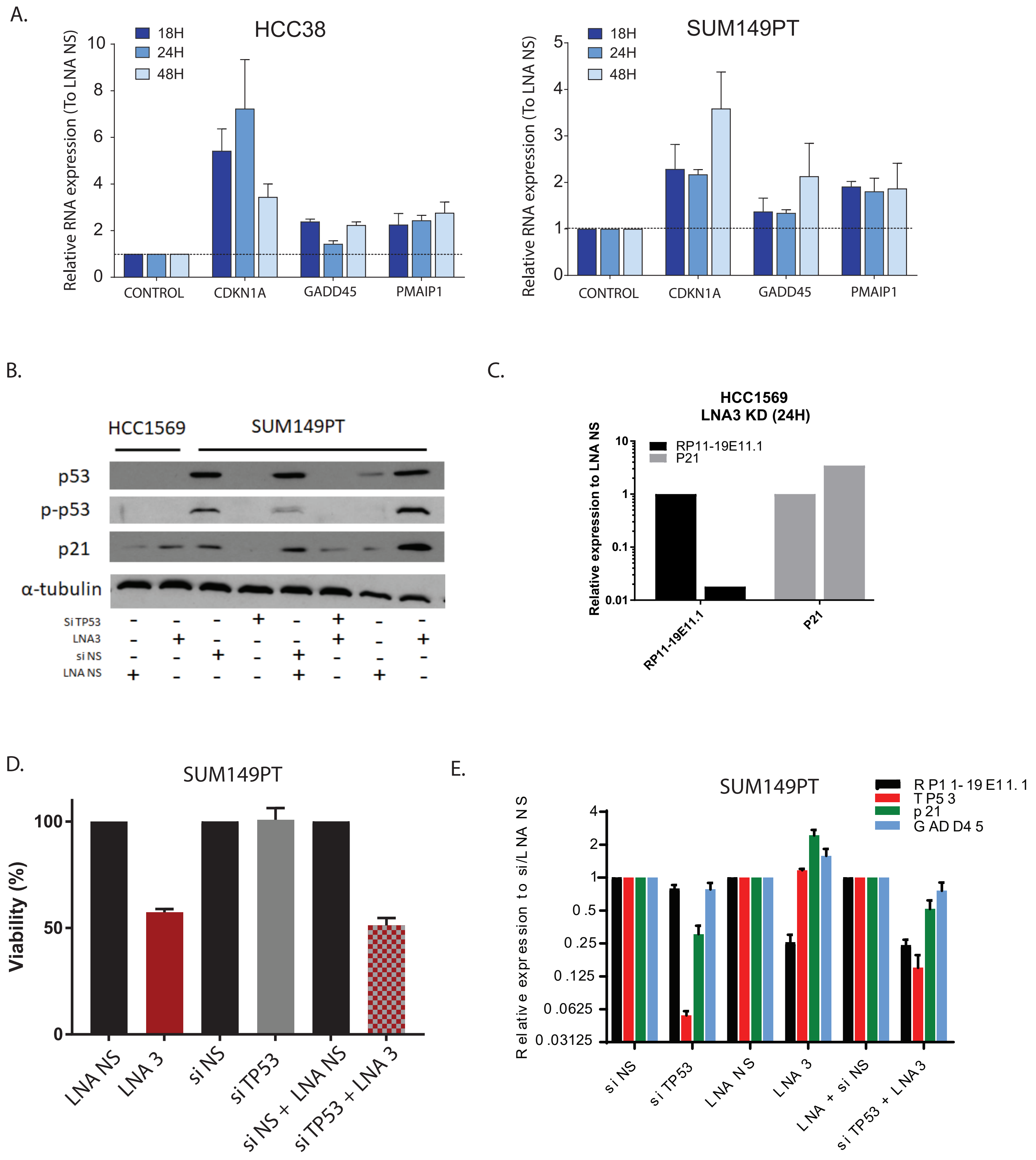

Supplementary Figure 5.Induction of P53 downstream genes **A)** P53 downstream genes levels after RP11-19E11.1 knock-down at different time points in two cell lines. **B)** Western-blot analysis of p53, p-p53 and p21 in the background of siP53, RP11-19E11.1 knock-down, or double knockdown. **C)** RNA levels of p21 in a P53 null cell line after RP11-19E11.1 knock-down. **D)** Viability assay (MTT) after 48h for the same conditions described before. **E)** RNA levels assessed by qRT-PCR of P53 canonical genes in the background of siP53, RP11-19E11.1 KD, or double P53 and RP11-19E11.1 knock-down.

Supplementary Figure 6

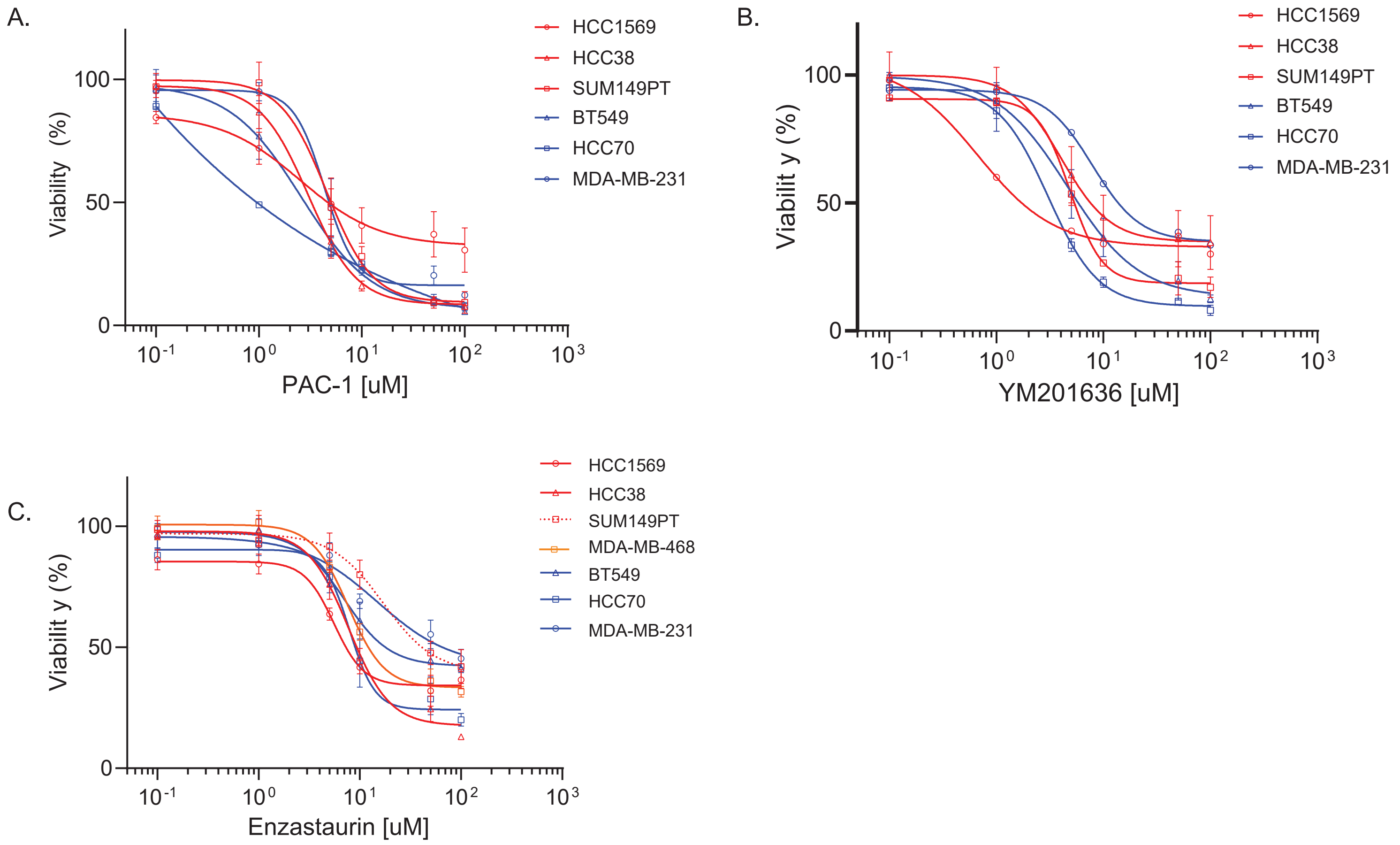

Supplementary Figure 6. Drug sensitivity screening. Results obtained for **A)** PAC-1 **B)** YM201636 and **C)** Enzastaurin results including the cell line SUM149PT Data are presented as mean ± SE.

Supplementary Figure 7

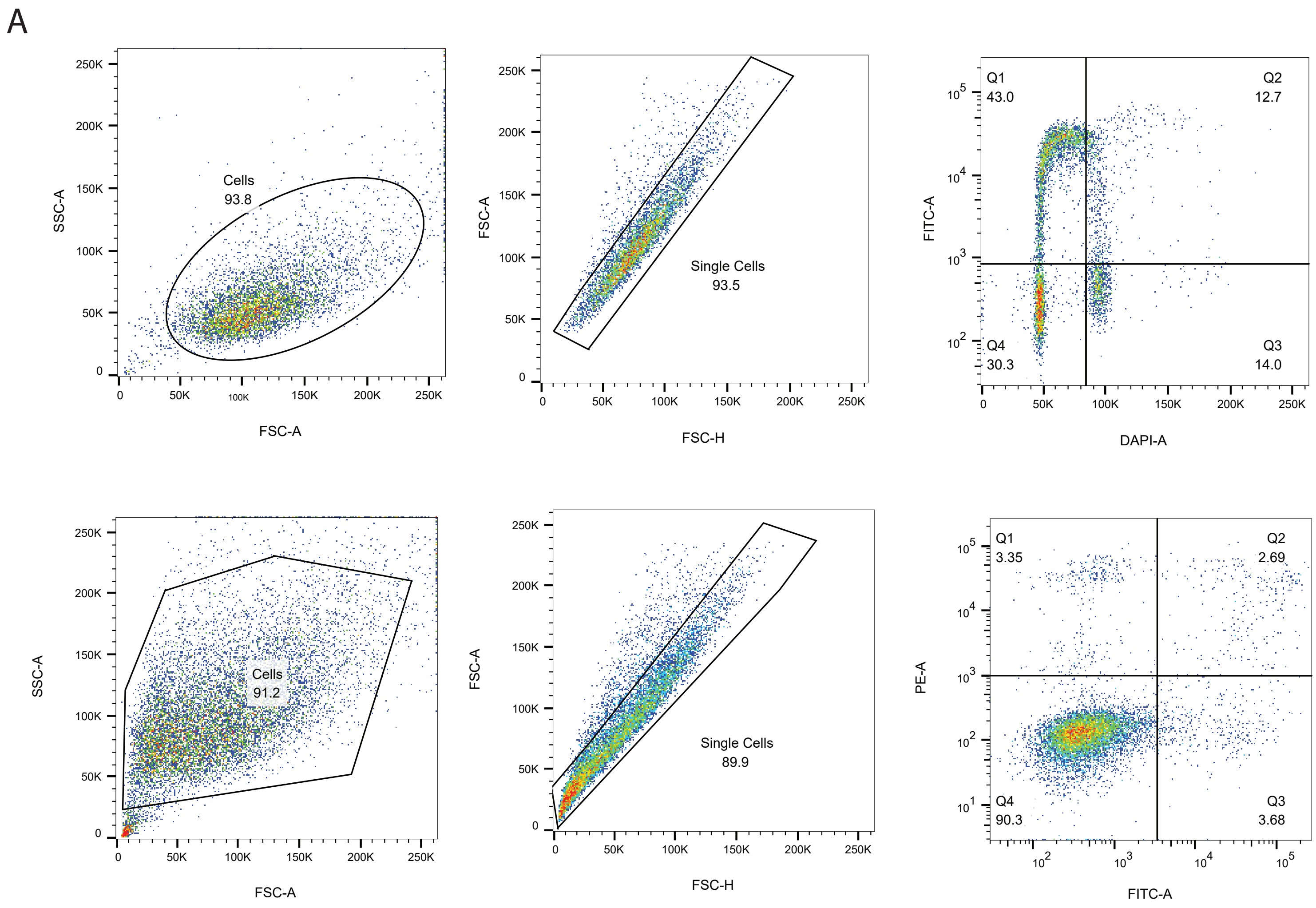

Supplementary Figure 7. FACs analysis. A) Gating example for EDU staining B) Gating example for Anexin-V.

Supplementary Methods

siRNA sequences (Dharmacon)

RP3-522D1.1_1	GCAGGCUCAAUCAAACCUUUU
RP3-522D1.1_2	GCUCAAUCAAACCUUCAAUUU
CTD-2015G9.2_1	CCGUGGAAACUGCAGACUUUU
CTD-2015G9.2_2	GCUGCAGAGGAGGGUGCUUUU
AC01917.2_1	UAGCAGAGUCAUACGGAAU
AC01917.2_2	GAUGAGGGCAUGUGGAGUU
AC01917.2_3	AGAUAAAGUAAACGUGGCA
AC01917.2_4	CCAGGCGGAGAGUGGACAA
EN1	ON-TARGETplus EN1 SMART pool #L-011266-00-0005

LNA GapmeR sequences (Qiagen)

LNA1 CGTGGTCCCGGAGAAA
LNA2 CGGAGAAAAGGAAGTG
LNA 3 GAATTTGGCACGGCGG

Primers for qRT-PCR

RP11-19E11.1 variant 1:
Fw: 5'-GGGCTGCCAACTGAACTTTT-3'
Rev: 5'-TTACATCGCAGTCCCCAAGT-3'

RP11-19E11.1 variant 2:
Fw: 5'-ATCCCTGCAGATTGAGCTCT-3'
Rev: 5'- ATCGCAGTCCCCAAGTCAA-3'

EN1
Fw: 5'-GCACACGTTATTCGGATCG-3'
Rev: 5'- GCTTGTCCTCCTTCTCGTTC-3'

GAPDH
Fw 5'-CGGAGTCAACGGATTTGGTCGTAT-3'
Rev: 5'- AGCCTTCTCCATGGTGGTGAAGAC-3'

GADD45
Fw 5'-CAGAAGACCGAAAGGATGGA-3'
Rev: 5'- AGTGATCGTGCGCTGACTC-3'

CDKN1A
Fw 5'-GGAAGACCATGTGGACCTGT-3'
Rev: 5'- GGATTAGGGCTTCCTCTTGG-3'

CTD-2015G9.2
Fw 5'-GCTGGTCAGTGTTTCCTCCT-3'
Rev: 5'- TCACAGGGGTGATCTCTGG-3'

AC01917.2
Fw 5'- CGAGCTCCACAAACTTCCAC-3'
Rev: 5'- CAGACAATACCTTCGGGCCT-3'

RP3-522D1.1
Fw 5'- CGGGAAAGAAGAGCAGGGTT-3'
Rev: 5'- AGTTGCTGGGTTTTCTGGCT-3'

PMAIP1
Fw 5'- GGACTGTTCGTGTTCAGCTC-3'
Rev: 5'- CACACTCGACTTCCAGCTCT-3'

TP53
Fw 5'- CAGCACATGACGGAGGTTGT-3'
Rev: 5'- TCATCCAAATACTCCACACGC-3'

SKP2

Fw 5’- CCCTGAGCTGCTAAAGGTCT-3’
Rev: 5’- GTGAGGTCTAAGGTCTGCCA-3’

E2F1
Fw 5'-AGCTGGACCACCTGATGATT-3'
Rv 5'-GGTCTGCAATGCTACGAAGG-3'

CCNE2
Fw 5'-ATACTGACTGCTGCTGCCTT-3'
Rv 5'-AGTCTTCAGCTTCACTGGACT-3'

MALAT-1
Fw 5'-GACGGAGGTTGAGATGAAGC-3'
Rv 5'-ATTCGGGGCTCTGTAGTCCT-3'

U1
Fw 5'-ATACTGACTGCTGCTGCCTT-3'
Rv 5'-CAGTCCCCCACTACCACAAATTA-3'

XIST
Fw 5'-ACGCTGCATGTGTCCTTAGTAGTC-3'
Rv 5'-ATTTGGAGCCTCTTATAGCTGTTTG-3'

NEAT-1
Fw 5'-CAGACCAAGGGCTGTGAACC-3'
Rv 5'-AAAAGGAGCACTGCCACCA-3'

h18S
Fw 5'-CGCCGCTAGAGGTGAAATTCT-3'
Rv 5'-CGAACCTCCGACTTTCGTTCT-3'

H7sk
Fw 5'-GACATCTGTCACCCCATTGA-3'
Rv 5'-GCCTCATTTGGATGTGTCTG-3'

RACE primers

3'RACE V1 5'-TGGGTGTTGGTGGAGGGGAGAATGG-3'
3'RACE V1_N 5'-GCTTGGGGTTAGGGAAAGGAGGCGC-3'
3'RACE V2 5'-CCCGCGCCTTCTATCCCTGCAGATT-3'
3'RACE V2_N 5'-GTCCGCCCAAGTGCCCTCCTCTCAG-3'
5'RACE 5'-CTTGACTTGCTAGGATTGGTGGGCC-3'
5'RACE_N 5'-CGTGGTCCCGGAGAAAAGGAAGTGA-3'

ChIP Primers

Chr7 neg Fw	5'-CTGTCACCATGCCCCACTA-3'
Chr7 neg Rev	5'-AATCGCTTCGTTGGTTTCAC-3'
CDC2 Fw	5'-CGCCCTTTCCTCTTTCTTTC-3'
CDC2 Rev	5'-ATCGGGTAGCCCGTAGACTT-3'

RP11-19E11.1 promoter region

Set 1
Fw 5'-CTTCTGCGACCAGCCTTCT-3'
Rv 5'-GTTCGGATGTCCAGTTGAGC-3'

Set 2
Fw 5'-GCTCAACTGGACATCCGAAC-3'
Rv 5'-TACTCGGAAGCCCAAGAGTC-3'

Set3
Fw 5'-CACCCCTGTATCCCTTTGA-3'

Rv 5'-GGTCTCTTCAGCCTTGCTGT-3'

Set4
Fw 5'-TGAGAGTAGCCACGCAATGT-3'
Rv 5'-GCCTCGTGTAACCATTCAGG-3'

Set5
Fw 5'-TGGTCCTGAATGGTTACACG-3'
Rv 5'-ATTTCAGAAGGGGCTCACCT-3'

Set6
Fw 5'-AGGTGAGCCCCTTCTGAAAT-3'
Rv 5'-GGGTAAGAGAACTGGCCACA-3'

Un-cropped images from Western Blot

Figure 5E

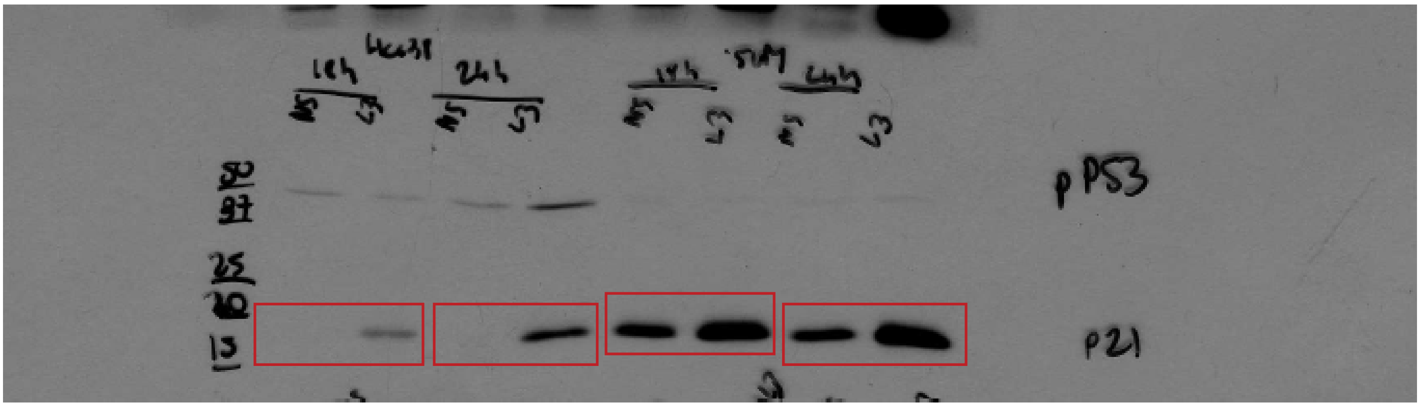

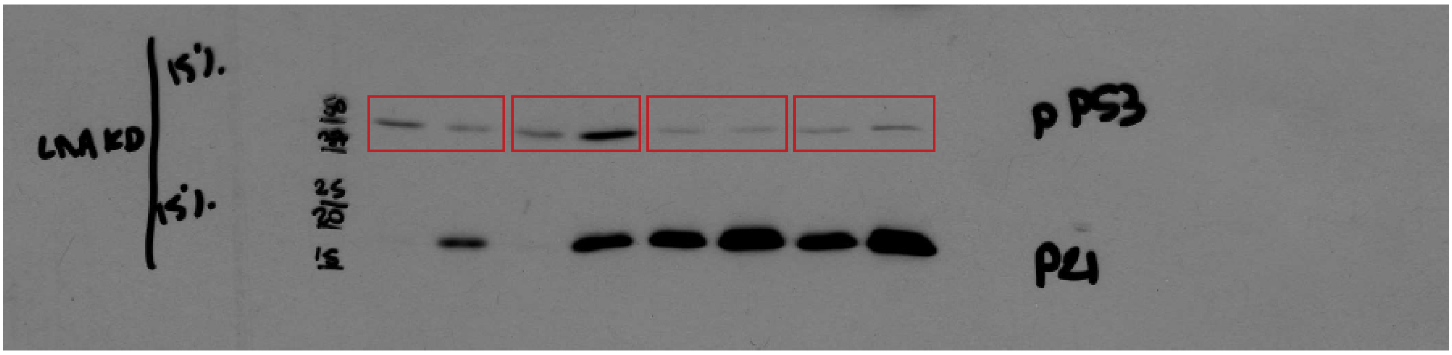

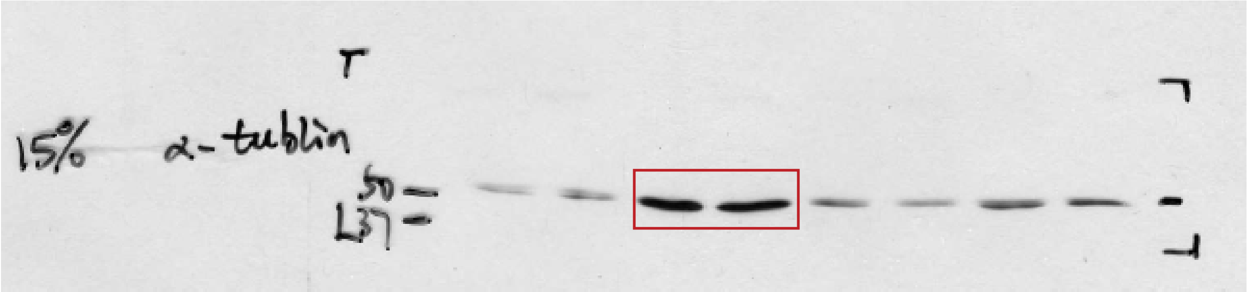

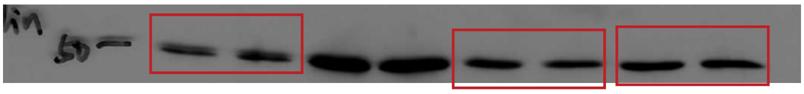

Figure 5E

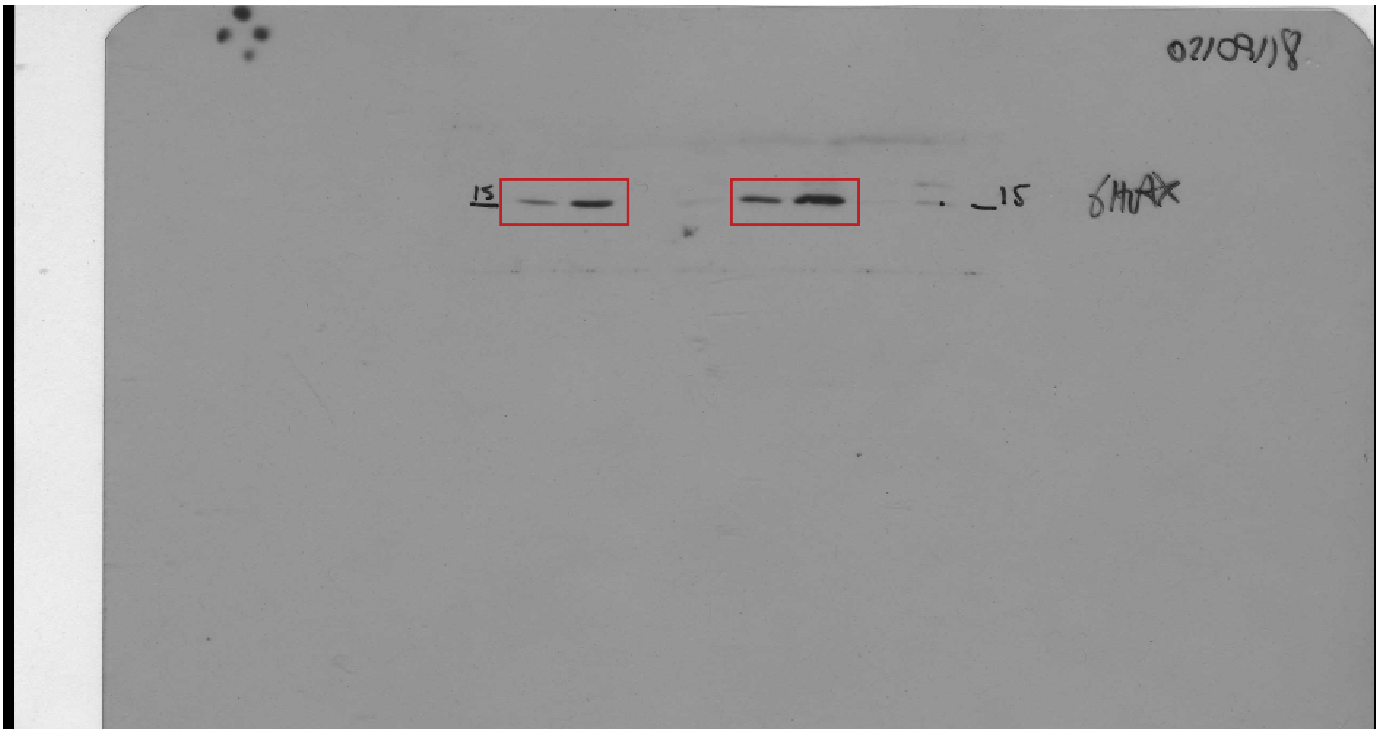

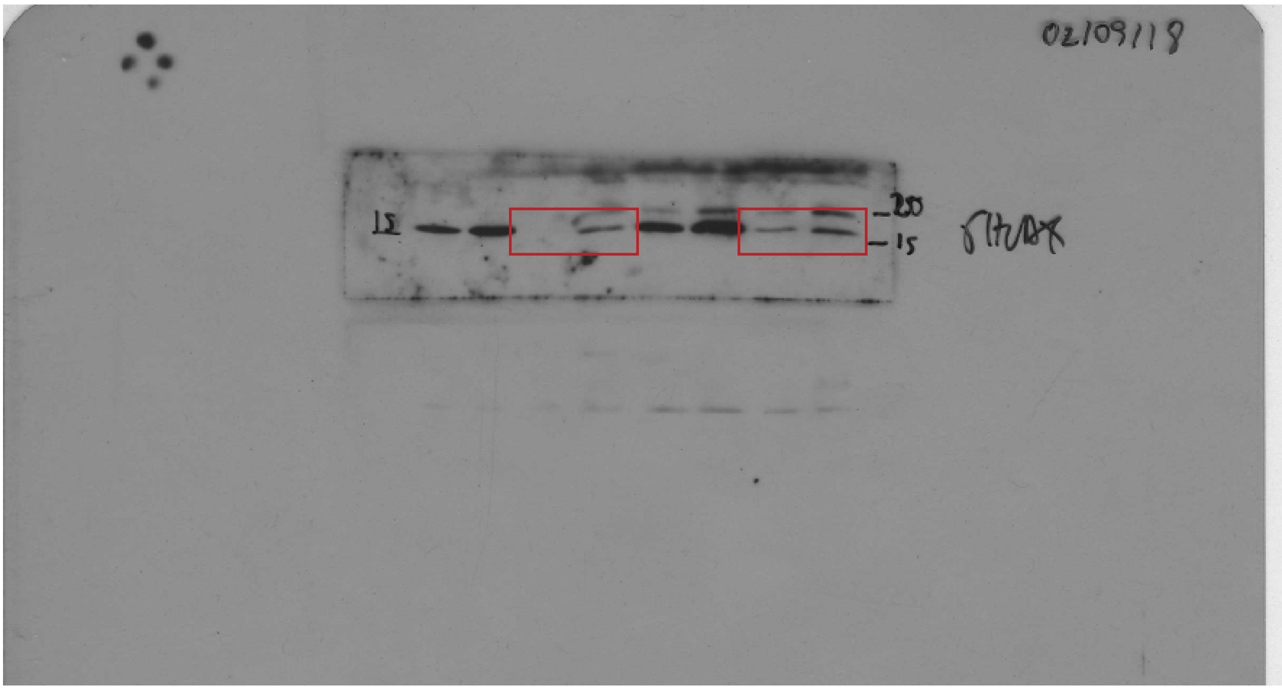

Figura 5E

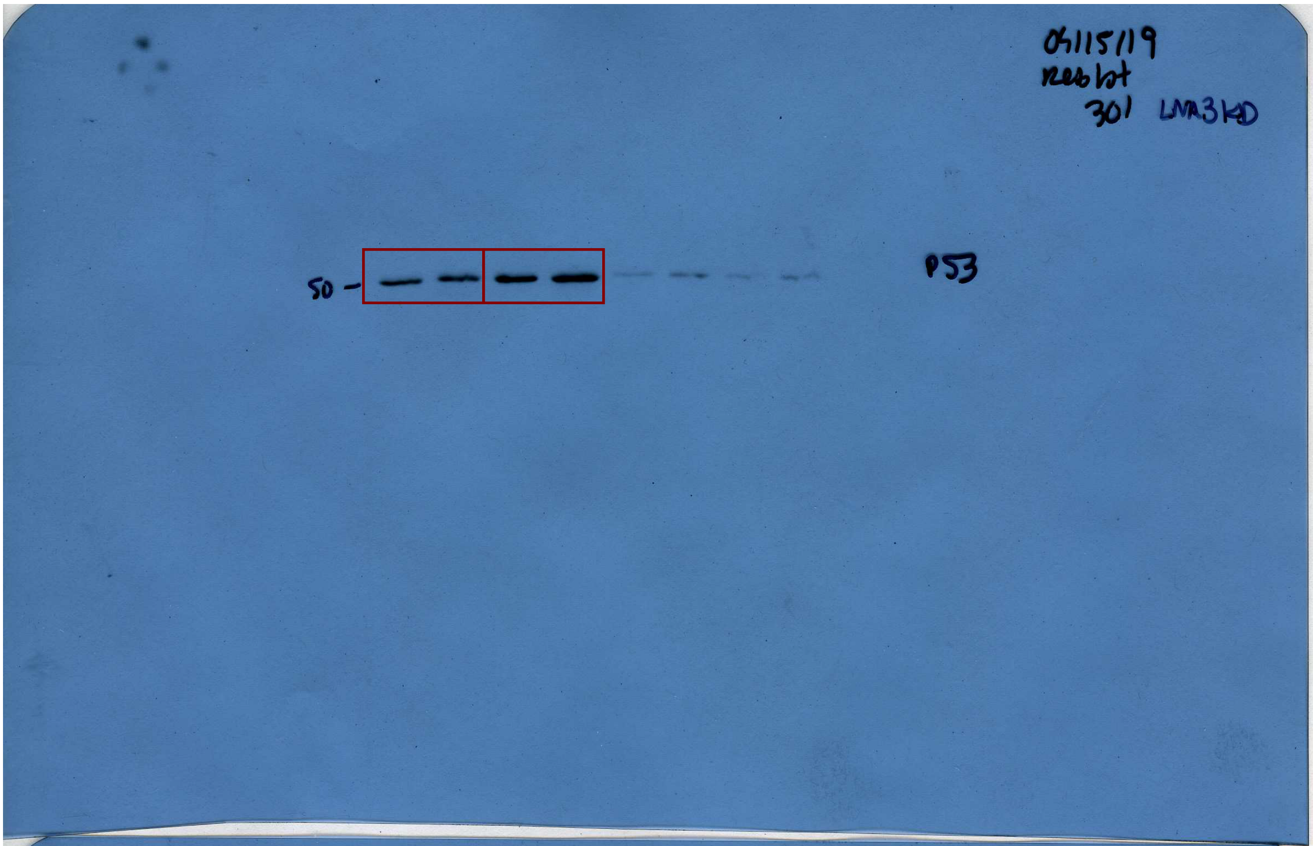
04/15/19
Reeblot
30' LMNB3 KD
50
P53

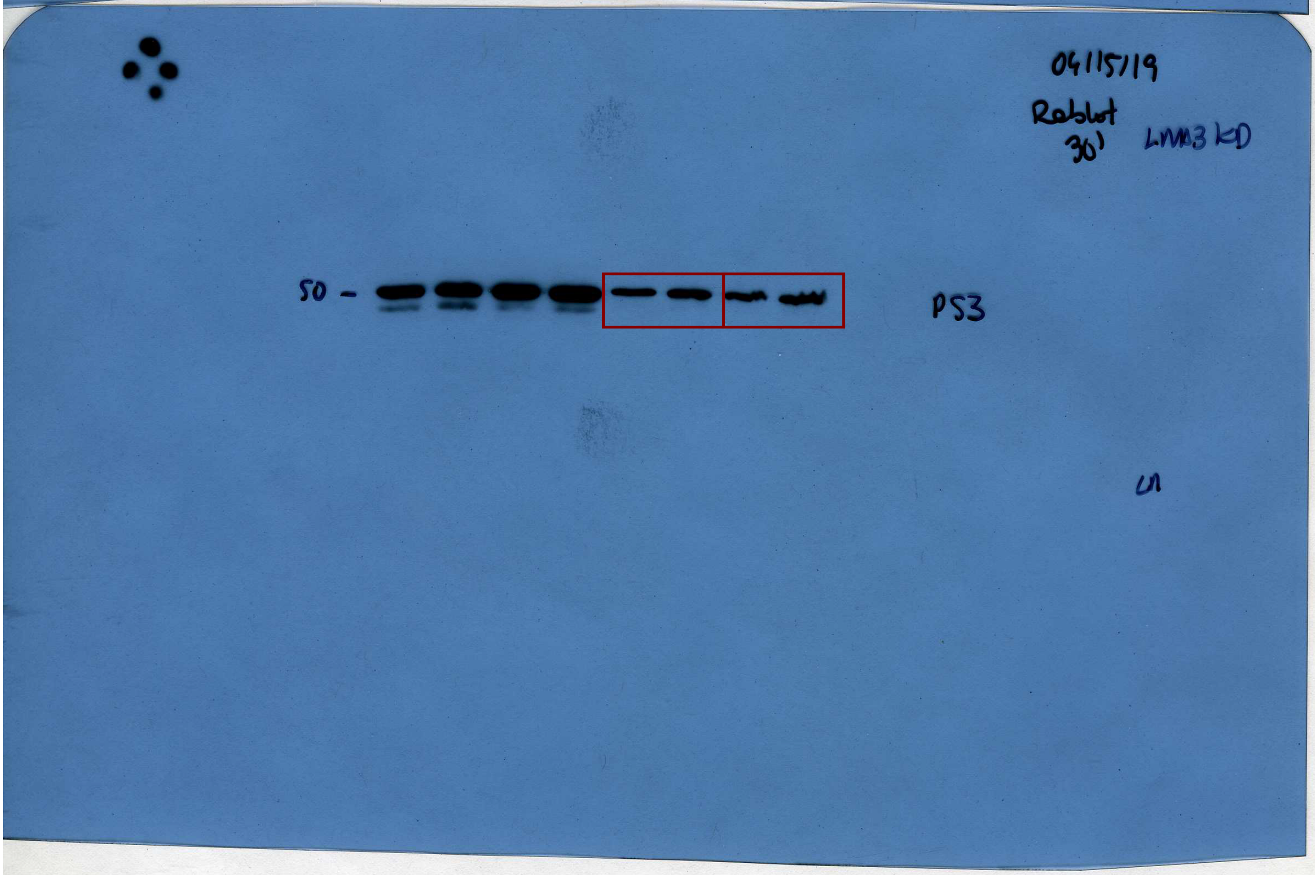
04/15/19
Reblot
30' LMNB3 KD
50
P53

Figure 6B

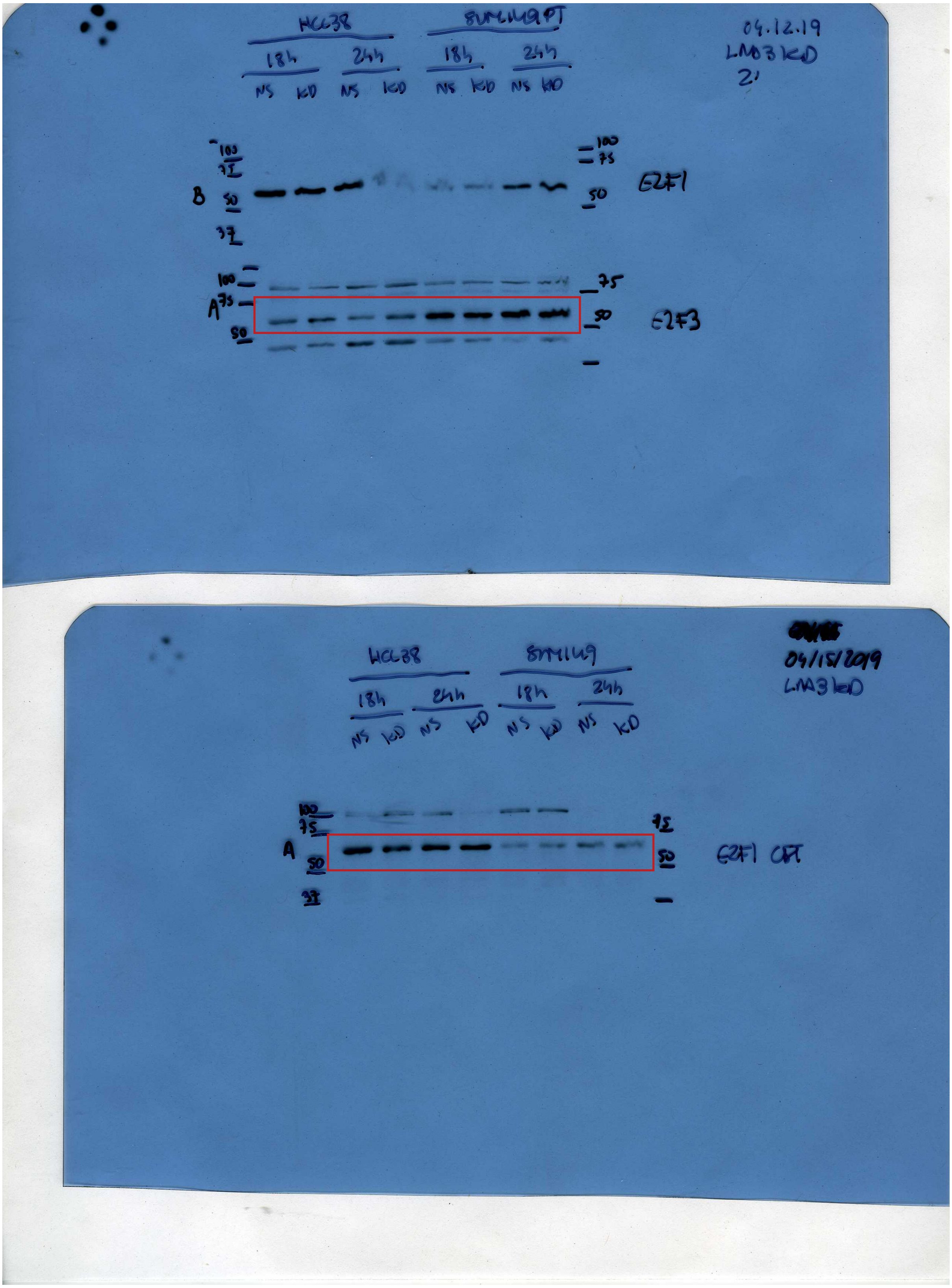

HCC38
SUM149PT
18h
24h
NS
KD
04.12.19
LMO3 KD
E2F1
E2F3
04/15/2019
E2F1

Figure 6B

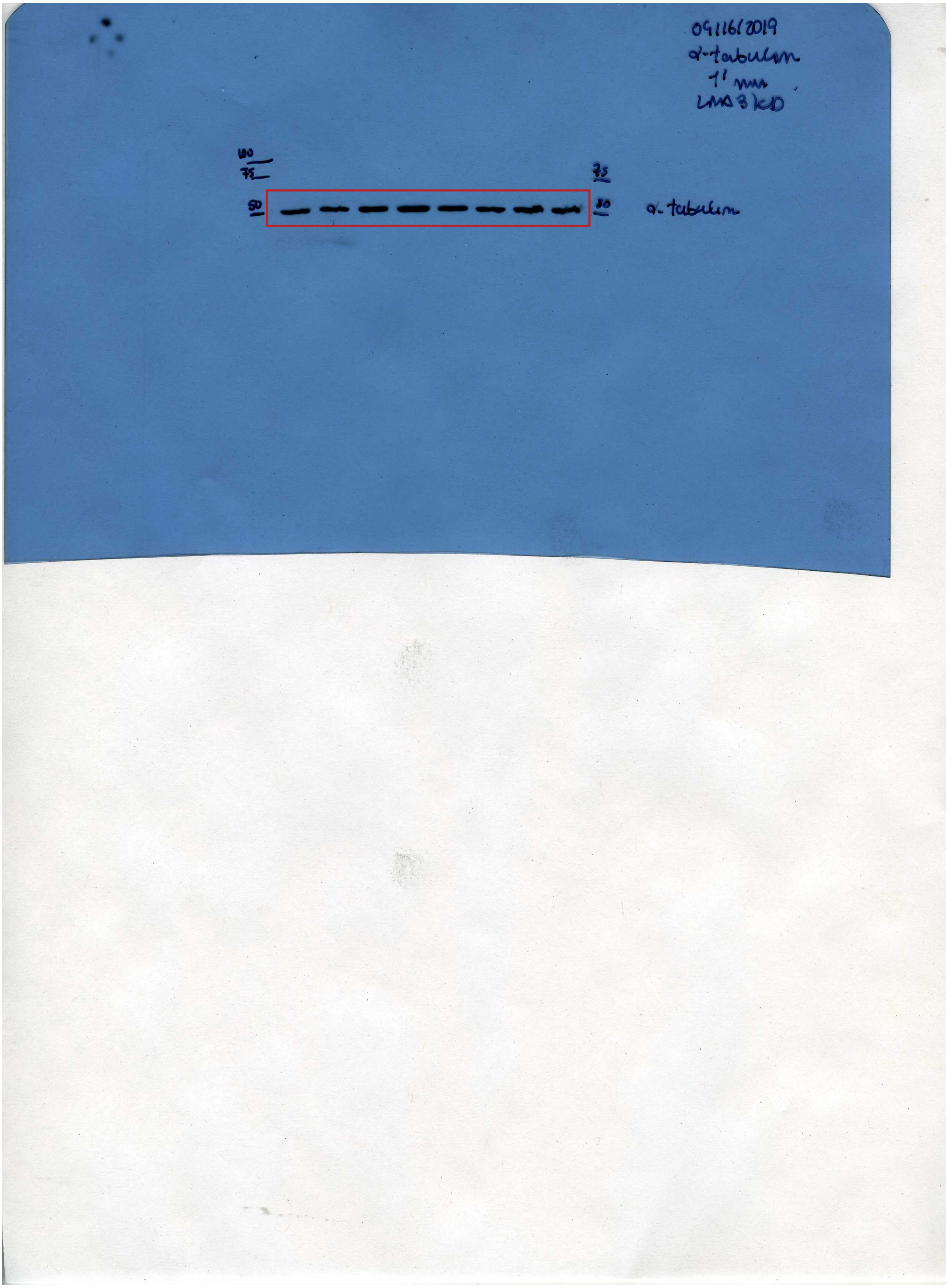

Supplement: Supplementary file 1 — Supplementary Information [file 41523_2019_144_MOESM1_ESM.pdf]
