# Supplementary material for: LncRNA RP11-19E11 is an E2F1 target required for proliferation and survival of basal breast cancer
Source: NPJ Breast Cancer. 2020 Jan 6;6:1. doi: 10.1038/s41523-019-0144-4 (PMC6944689; doi:10.1038/s41523-019-0144-4)
Supplement: Supplementary file 2 — Reporting Summary Checklist [file 41523_2019_144_MOESM2_ESM.pdf]

nature research

Corresponding author(s): Francisco J. Esteva
Irma Sanchez

Last updated by author(s): 09/25/2019

Reporting Summary

Nature Research wishes to improve the reproducibility of the work that we publish. This form provides structure for consistency and transparency in reporting. For further information on Nature Research policies, see Authors & Referees and the Editorial Policy Checklist.

Statistics

For all statistical analyses, confirm that the following items are present in the figure legend, table legend, main text, or Methods section.

n/a	Confirmed	
☐	☒	The exact sample size (*n*) for each experimental group/condition, given as a discrete number and unit of measurement
☐	☒	A statement on whether measurements were taken from distinct samples or whether the same sample was measured repeatedly
☐	☒	The statistical test(s) used AND whether they are one- or two-sided<br>*Only common tests should be described solely by name; describe more complex techniques in the Methods section.*
☒	☐	A description of all covariates tested
☐	☒	A description of any assumptions or corrections, such as tests of normality and adjustment for multiple comparisons
☐	☒	A full description of the statistical parameters including central tendency (e.g. means) or other basic estimates (e.g. regression coefficient) AND variation (e.g. standard deviation) or associated estimates of uncertainty (e.g. confidence intervals)
☐	☒	For null hypothesis testing, the test statistic (e.g. *F*, *t*, *r*) with confidence intervals, effect sizes, degrees of freedom and *P* value noted<br>*Give P values as exact values whenever suitable.*
☒	☐	For Bayesian analysis, information on the choice of priors and Markov chain Monte Carlo settings
☒	☐	For hierarchical and complex designs, identification of the appropriate level for tests and full reporting of outcomes
☒	☐	Estimates of effect sizes (e.g. Cohen's *d*, Pearson's *r*), indicating how they were calculated

Our web collection on statistics for biologists contains articles on many of the points above.

Software and code

Policy information about availability of computer code

Data collection	The read count tables for all the sequencing samples (n=1162) in TCGA were downloaded from The National Cancer Institute's (NCI) Genomic Data Commons (GDC). All the raw sequencing reads from our samples and the cell lines downloaded from GSE73526 and GSEA48213.
Data analysis	TCGA data<br>The count tables were normalized based on their library size factors using DEseq2, and differential expression analysis was performed. Clustering was performed using iCellR (https://github.com/rezakj/iCellR) by selecting the dispersed genes running PCA and tSNE. All the methylation Beta values for the samples with Illumina 450 methylation array data (n=892) were downloaded from Genomic Data Commons (GDC).<br>RNA-seq<br>All the raw sequencing reads from our samples and the cell lines downloaded were mapped to the human reference genome (GRCh37/hg19) using the STAR aligner (v2.5.0c) (Dobin et al., 2013). Alignments were guided by a Gene Transfer Format file. The mean read insert sizes and their standard deviations were calculated using Picard tools (v.1.126) (http://broadinstitute.github.io/picard). The read count tables were generated using HTSeq (v0.6.0) (Anders et al., 2015) normalized based on their library size factors using DEseq2 (Love et al. 2014), and differential expression analysis was performed. The Read Per Million (RPM) normalized BigWig files were generated using BEDTools (v2.17.0) (Quinlan and Hall, 2010) and bedGraphToBigWig tool (v4). All downstream statistical analyses and generating plots were performed in R environment (v3.1.1) (http://www.r-project.org/).

For manuscripts utilizing custom algorithms or software that are central to the research but not yet described in published literature, software must be made available to editors/reviewers. We strongly encourage code deposition in a community repository (e.g. GitHub). See the Nature Research guidelines for submitting code & software for further information.

Data

Policy information about availability of data

All manuscripts must include a data availability statement. This statement should provide the following information, where applicable:

- Accession codes, unique identifiers, or web links for publicly available datasets
- A list of figures that have associated raw data
- A description of any restrictions on data availability

The RNA-seq raw data generated in this study is deposited in the Gene Expression Omnibus (GEO) database with the accession number #TBD. Figures associated with this raw data are Figure 3E, Figure 5C and D, Figure 6A.

Field-specific reporting

Please select the one below that is the best fit for your research. If you are not sure, read the appropriate sections before making your selection.

☒ Life sciences ☐ Behavioural & social sciences ☐ Ecological, evolutionary & environmental sciences

For a reference copy of the document with all sections, see nature.com/documents/nr-reporting-summary-flat.pdf

Life sciences study design

All studies must disclose on these points even when the disclosure is negative.

Sample size	This study was based in the analysis of TCGA data from (882) 769 patients represented by 131 Basal-like, 64 HER2, 404 Luminal A, 170 Luminal B, 25 Normal-like subtypes and 113 samples of Normal-Tissue.
Data exclusions	TCGA patients with unknown molecular classification (no PAM50) were excluded from the analysis.
Replication	All experiments were performed at least two or three times in duplicate/triplicate.
Randomization	Not relevant to our study
Blinding	Not relevant to our study

Reporting for specific materials, systems and methods

We require information from authors about some types of materials, experimental systems and methods used in many studies. Here, indicate whether each material, system or method listed is relevant to your study. If you are not sure if a list item applies to your research, read the appropriate section before selecting a response.

Materials & experimental systems

n/a	Involved in the study	
☐	☒	Antibodies
☐	☒	Eukaryotic cell lines
☒	☐	Palaeontology
☒	☐	Animals and other organisms
☒	☐	Human research participants
☒	☐	Clinical data

Methods

n/a	Involved in the study	
☒	☐	ChIP-seq
☐	☒	Flow cytometry
☒	☐	MRI-based neuroimaging

Antibodies

Antibodies used	The primary antibody used and dilutions are α-tubulin (Sigma T5168, 1:5000), E2F1 (CST #3742, 1:1000), E2F3 (Gentex GTX11843, 1:2000), p21 (CST #2947, 1:1000), P53 (Santa Cruz FL-393, 1:1000), p-P53 (CST #9286, 1:1000), γH2AX (CST #9718, 1:1000).
Validation	All antibodies are commercially available and have been validated by the company or numerous research articles.

Eukaryotic cell lines

Policy information about cell lines

Cell line source(s)	MDA-MB-231, Hs578T, MDA-MB-157, MDA-MB-468, MCF7, SkBr3, BT474, HCC2157, HCC38, HCC2157, BT549, HCC70,

Cell line source(s)	HCC1143, T-47D were obtained from ATCC. SUM149PT, SUM159PT were a gift from Neel BG laboratory (Cell. 2016 Jan 14;164(1-2):293-309. doi: 10.1016/j.cell.2015.11.062.).
Authentication	Non of the cells lines have been authenticated.
Mycoplasma contamination	All the cells lines are Mycoplasma negative. Periodic test using ATCC PCR Mycoplasma detection kit have been performed.
Commonly misidentified lines (See ICLAC register)	Not relevant to our study

Flow Cytometry

Plots

Confirm that:

☒ The axis labels state the marker and fluorochrome used (e.g. CD4-FITC).

☒ The axis scales are clearly visible. Include numbers along axes only for bottom left plot of group (a 'group' is an analysis of identical markers).

☒ All plots are contour plots with outliers or pseudocolor plots.

☒ A numerical value for number of cells or percentage (with statistics) is provided.

Methodology

Sample preparation	For EdU and cell cycle analysis, Click-iT EdU Alexa Fluor 488 kit was used (Invitrogen C10337) following manufacturer’s instructions.<br>Annexin-V staining was performed using Annexin-V Kit (Mileny Biotec, 130-092-052) following manufacturer instructions.
Instrument	All analyses were performed with LSRII UV cell analyzer (BD bioscience).
Software	All analyses were performed with FlowJo software.
Cell population abundance	All all our analysis are from a homogeneous population (established breast cancer cell lines), so after sorting out doublets, the remain analysis include 85-99% of the cells, containing at least 5000 single cells.
Gating strategy	We selected our population first screening FSC/SCC. Then, to exclude doublets, we plotted FSC-H/FSC-A. Single cell population was selected for further analysis and plotted DAPI/EDU-Alexa468 or PI/ANEXINV-FITC. Bounders were selected using negative controls (untreated cells as reference).

☒ Tick this box to confirm that a figure exemplifying the gating strategy is provided in the Supplementary Information.
